# Insights into bone and cartilage responses to pulsed electromagnetic field stimulation: a review with quantitative comparisons

**DOI:** 10.3389/fbioe.2025.1557572

**Published:** 2025-07-10

**Authors:** Beatrice Masante, Stefano Gabetti, Joao C. Silva, Giovanni Putame, Simone Israel, Cristina Bignardi, Diana Massai

**Affiliations:** ^1^ Department of Mechanical and Aerospace Engineering and Polito^BIO^Med Lab, Politecnico di Torino, Turin, Italy; ^2^ Interuniversity Center for the Promotion of the 3Rs Principles in Teaching and Research, Turin, Italy

**Keywords:** pulsed electromagnetic field (PEMF), bone, cartilage, bone fractures, osteoporosis, osteoarthritis, fracture healing, quantitative comparison

## Abstract

Bone fractures and cartilage pathologies represent a heavy socioeconomic burden for the national healthcare systems worldwide. Pulsed electromagnetic field (PEMF) stimulation has become a widely recognized treatment for enhancing bone fracture healing and reducing tissue inflammation, thereby supporting bone tissue regeneration. More recently, its effectiveness in treating cartilage degeneration and osteoarthritis has also been demonstrated. However, the effects of PEMF, particularly the underlying mechanisms related to the activation of specific signaling pathways, are not yet fully known neither correlated with the specific PEMF parameters applied. As a result, standardized protocols for PEMF treatment are lacking in clinical practice, leading to empirical application of PEMF stimulation and heterogeneity in treatment protocols. For these reasons, over the past three decades, the biological effects of PEMF on bone and cartilage tissues have been extensively investigated through both *in vitro* and *in vivo* experiments. The aim of this review is to provide a detailed overview of the performed studies, focusing on the applied PEMF stimulation parameters and the induced effects on bone and cartilage tissues. Furthermore, to enable comparisons across various published protocols and to aid in understanding the correlation between applied PEMF parameters and their resulting biological effects, we propose, for the first time, a quantitative descriptor for PEMF stimulation, termed PEMF dose, which accounts for magnetic field intensity, stimulation waveform, and exposure duration. The use of this comprehensive descriptor enabled the identification of common features across different studies and, in the future, it could serve as a valuable tool for refining PEMF stimulation protocols and establishing standardized guidelines to support bone and cartilage repair.

## 1 Introduction

Bone fragility fractures and osteoarthritis (OA) are two of the most prevalent disorders and among the primary causes of disability worldwide, with a significant impact on the socio-economic landscape (Borgström et al., 2020; Wu et al., 2021). In 2019, considering the European Union, Switzerland, and the United Kingdom, 25.5 million women and 6.5 million men over 50 years of age were estimated to have osteoporosis, with total direct costs associated with osteoporotic fractures accounting for € 57 billion. Moreover, 4.3 million new osteoporosis-related fractures were registered in 2019, with an expected increase of 25% in the number of fractures by 2034 (Kanis et al., 2021; Willers et al., 2022; IOF, 2024). Concerning OA, it was estimated that it afflicts over 500 million individuals worldwide (Hunter et al., 2020), with estimates indicating that, by 2032, 45% of adults over the age of 45 will be affected by OA (Miguel et al., 2022). These projections can be attributed to several interactive factors, primarily population aging combined with the rising prevalence of risk factors such as obesity, sedentary lifestyle, tobacco consumption (Amini et al., 2013; Suryani et al., 2019), and the prolonged use of medications known to affect musculoskeletal health, including glucocorticoids, certain anticonvulsants, and hormonal contraceptives (Ampatzis et al., 2022).

Although bone has an intrinsic capacity for regeneration and functional recovery through self-healing mechanisms (Salhotra et al., 2020; Varani et al., 2021), certain fractures exhibit delayed healing or complications such as non-unions, often resulting in chronic pain and significant impairment (Freeman et al., 2015). In contrast, cartilage injuries do not undergo spontaneous regeneration due to the avascularity and complex zonal architecture of the tissue (Gu et al., 2023). The gold standard interventions for repairing bone and cartilage defects—particularly in cases of trauma or sports injuries—rely on surgical procedures involving the implantation of autografts or allografts, frequently supplemented with biophysical therapies. Nonetheless, these approaches are associated with clinical limitations, including donor site morbidity in autografts and immunogenic or infectious risks in allografts (Bentley et al., 2012; Gaspar et al., 2012). In the context of osteoarthritis (OA), however, therapeutic strategies vary according to disease stage, with early management typically centered on pharmacological pain control and lifestyle modifications, while surgical interventions such as microfracture or joint replacement are considered for advanced stages (McAlindon et al., 2014).

Beside conventional surgical procedures, bone and cartilage tissue engineering (TE) approaches are emerging as promising strategies to promote the healing process (Daher et al., 2009; Amini et al., 2013; Langer and Vacanti, 2016; Pedrero et al., 2021; Miguel et al., 2022). Ideally, TE aims to generate three-dimensional (3D) functional substitutes for implantation, designed to mimic the architecture, composition, biology, and mechanical cues of the native tissue. However, the clinical translation of TE approaches remains constrained by scientific, technical, and regulatory challenges (Davies et al., 2014; Mittwede et al., 2018). As a result, TE constructs are primarily used as biomimetic tissue models for *in vitro* research and pre-clinical studies (Quarto and Giannoni, 2016). Moreover, for advancing the understanding of tissue behavior, significant progress has been made in developing bioreactors for biophysical stimulation, enabling the investigation of cellular and tissue responses to physical stimuli (Lanza et al., 2000; Massai et al., 2013). When cultured within bioreactors, biomimetic tissue models can be studied in a monitored and controlled environment, allowing for the exploration of complex cellular behaviors under various biophysical conditions. Over the past decade, several *in vitro* models and bioreactors have been used to investigate the influence of different physical stimulations—including mechanical loading (Hoenig et al., 2011; Matziolis et al., 2011; Hoffmann et al., 2015; Ravichandran et al., 2017; Lovecchio et al., 2019), fluid shear stress (Bancroft et al., 2002; Wendt et al., 2003; Datta et al., 2006; Du et al., 2009; Li et al., 2009; da Silva et al., 2010; Kavlock and Goldstein, 2011; Beşkardeş et al., 2018; Engel et al., 2021; Gabetti et al., 2022; Yamada et al., 2022), ultrasounds (Claes and Willie, 2007; Lim et al., 2013; Zhou X. et al., 2016; Ricotti et al., 2024), and electrical fields (Wiesmann et al., 2001; Dauben et al., 2016; Jing et al., 2019; Leppik et al., 2019; Portan et al., 2019)—on bone and cartilage tissues. Through these studies, researchers have gained valuable insights into how specific biophysical stimulations can enhance cellular responses, such as osteogenic and chondrogenic differentiation, matrix mineralization, and tissue remodeling, with the final aim to optimize the biophysical treatments.

Among the biophysical stimulations explored to promote bone and cartilage healing, electrical and electromagnetic fields have been used in clinical settings for over 40 years; however, detailed characterization of the signaling pathways they activate has become the subject of intense investigation only recently. Indeed, since bone and cartilage are characterized by piezoelectric properties (Jacob et al., 2018)—meaning that mechanical deformations generate electrical polarization in these tissues, known to play a key role in maintaining or repairing tissue (Fukada and Yasuda, 1957)—it has been hypothesized that external electrical or electromagnetic fields can enhance the body’s natural repair mechanisms (Barker and Lunt, 1983). For the application of these stimulations, three main techniques have been proposed (Kuzyk and Schemitsch, 2009; Leppik et al., 2020): direct current (DC), capacitive coupling (CC), and inductive coupling (IC). In DC stimulation, electrodes are placed in direct contact with the tissue to generate an electric current. This invasive method is commonly employed in research and clinical settings, but it is susceptible to complications, including issues related to electrode insertion, electrode corrosion, and the release of metallic ions. CC represents a non-invasive alternative, in which the target tissue is placed between two electrodes that are in mechanical contact with the skin but electrically insulated, avoiding flow of direct current between the electrodes and the tissue. However, achieving the same level of effectiveness as DC typically requires the application of higher voltages. Alternatively, IC offers a further non-invasive option that employs pulsed electromagnetic fields (PEMF). The PEMF stimulation is obtained by imposing an alternating current along a single solenoid or a pair of Helmholtz coils and positioning the target near the solenoid or between the coils. The alternating current generates a time-varying magnetic field that induces a time-varying electric field in the tissue, as demonstrated by Faraday and Lenz. The magnitude and frequency of the induced electric field are dependent on the variation of the magnetic flux in the tissue and on tissue properties. IC mode operates at low frequencies (6–500 Hz) and low intensities (0.1–30 mT), making it less energy-intensive than CC and more suitable for clinical and home use (Hu et al., 2020).

The main effect of such stimulations is to induce a time-varying electric field in the exposed tissue, like the one naturally generated during movement (Varani et al., 2021). This secondary electric field depolarizes cell membranes, initiating ionic currents that activate signaling pathways responsible for tissue regeneration (Markov, 2007). Since the Food and Drugs Administration (FDA) approval in 1979 for the treatment of non-union fractures, backed by pioneering studies (Andrew et al., 1974; Bassett et al., 1977; Friedenberg and Brighton, 1974; Brighton et al., 1975), PEMF stimulation has gained increasing importance in orthopedic clinical practice (Nelson et al., 2013). Several studies demonstrated its potential in enhancing bone endogenous healing processes (Chalidis et al., 2011; Daish et al., 2018; Cadossi et al., 2020) and for the prevention and treatment of osteoporosis (Wang et al., 2021), osteonecrosis (Massari et al., 2006) and OA (Veronesi et al., 2014). Moreover, several clinical trials have shown the efficacy of PEMF therapy in the treatment of numerous conditions, including non-union bone fractures (NCT01574833 (XIONG, 2012)), osteoporosis (NCT04608162 (El-Shamy, 2020)), and OA (NCT04106986 (Jordan University of Science and Technology, 2021); NCT01877278 (Bagnato et al., 2016)).

Indeed, clinical studies performed using different devices showed biological effectiveness when applying PEMF with exposure times of 3–8 h per day from 3 to 10 months to treat fracture non-unions or pseudoarthrosis (Garland et al., 1991; Massari et al., 2006; Cebrián et al., 2010; Faldini et al., 2010). However, due to the different device operating parameters—such as intensity, frequency, waveform—standardized guidelines have not been defined yet (Hu et al., 2020). Furthermore, although numerous PEMF stimulation devices are commercially available and widely used in clinical practice, it is important to note that the European regulation for the market authorization of medical devices has long required only the verification of electrical safety, with the risk of exposing patients to ineffective devices. It was only in 1993 that the European directive came into effect, mandating manufacturers to provide evidence demonstrating the effectiveness of medical devices before they could be commercialized (Massari et al., 2011). Given the heterogeneity of parameters used, the regulatory requirements, and the consistently positive effects reported in literature, further research on PEMF stimulation is essential, together with strategies for enabling comparability.

This review aims to illustrate the advancements made over the past three decades in understanding the effects of PEMF stimulation on bone and cartilage tissues, with a particular focus on findings from both *in vitro* and *in vivo* studies. Moreover, we introduced, for the first time, the quantitative descriptor PEMF dose. This is a comprehensive metric based on PEMF stimulation parameters (i.e., magnetic field intensity, stimulation waveform, and exposure duration) which was crucial for comparing the protocols and outcomes obtained using different PEMF stimulators and applying different operating parameters and for identifying quantitative thresholds for biological responses.

## 2 Background to composition and electroactive properties of bone and articular cartilage tissues

Bone and articular cartilage are connective tissues composed predominantly by their respective resident cells embedded in a dense extracellular matrix (ECM). While articular cartilage is uniquely composed of chondrocytes, bone tissue resident cells include osteoblasts (bone ECM production), osteoclasts (bone resorption), osteocytes (terminally differentiated mature bone cells and main mechanoreceptors of the tissue), and bone lining cells (Schaffler and Kennedy, 2012). For both bone and cartilage tissues, collagen is the main constituent of their ECM. Once the collagen fibers are exposed to mechanical stress due to daily movement, its macromolecules, which are highly composed by glycine, proline and hydroxyproline residues (with NH and CO units), experience reorientation towards the protein long axis and magnitude change of their dipole moment (Zhou Z. et al., 2016). Such responses to mechanical stimuli are responsible for the intrinsic piezoelectricity of collagen, which plays a crucial role in influencing the endogenous bioelectric signaling and electrical features of bone and cartilage tissues (Chen et al., 2024). However, bone and cartilage respond differently to PEMF stimulation due to their distinct resident cell populations, biological functions, composition, structure, and mechanical and electroactive properties (summarized in Table 1). The following sections offer insights into the composition and associated electroactive properties of bone and cartilage tissues.

**TABLE 1:** Summary of the main differences observed between bone and cartilage tissues in terms of their response to PEMF stimulation.

Property	Bone	Cartilage
Main cell populations responsive to PEMF	Osteoblasts, MSCs, osteoclasts	Chondrocytes, chondroprogenitor cells, MSCs
Electroactive properties	Intrinsically piezoelectric and dielectric material	Electrokinetic properties
Tissue composition	Mineralized matrix, rich in collagen type I, highly vascularized	Avascular, soft matrix (mainly composed by collagen type II and proteoglycans)
Tissue regenerative capacity	High, self-healing capacity for small defects, rapid healing (enhanced with PEMF)	Limited, enhanced with PEMF but requires additional support (e.g., chemical cues)
Reported overall PEMF effects	Promotes osteogenesis, matrix mineralization, enhances bone formation *in vivo*, fracture healing	Promotes chondrocyte proliferation, chondrogenesis, cartilage ECM production, *in vivo* cartilage repair

### 2.1 Bone

Bone tissue is considered a composite material consisting of an inorganic (65%) and organic (35%) phase. The bone organic phase consists mainly (90%) in collagen type I (COL1) organized in fibrils, providing the tissue its high tensile mechanical strength. The inorganic phase consists mainly in calcium phosphate in the form of hydroxyapatite nanocrystals deposited within the collagen fibrils, being responsible for mineral exchange and for the high compressive strength (Baxter et al., 2010; Domingues et al., 2024). Macroscopically, bone tissue can be categorized into two main types: compact (also known as cortical) bone and cancellous (also known as spongy or trabecular) bone. Compact bone constitutes approximately 80% of the human skeleton, serving as a hard, dense protective outer layer that envelops long bones throughout the body. Cancellous bone (20%), which is less dense (high porosity – 50%–90%) than the compact bone and includes the inner layer of irregular bones, contributes to the load absorption and metabolic exchange capacities of the tissue (Collins et al., 2021). Moreover, cancellous bone comprises most of the human body’s bone marrow, which is responsible for the production of blood cells and harbors many stem cells involved in bone repair and remodeling processes. In particular, the human bone marrow-derived stem/stromal cells (hBMMSCs) have been considered the “gold standard” cell source for cell therapy and TE strategies for bone regeneration, mainly due to their superior tissue-specific potential for osteogenesis, high *in vitro* proliferation capacity, and advantageous trophic/immunomodulatory properties (Silva et al., 2021; Zha et al., 2022; Rossi et al., 2023).

The dielectric and piezoelectric features of natural bone, responsible for the bioelectric signaling occurring within the tissue regulating its remodeling and homeostasis, have been previously demonstrated (Wang et al., 2023; Chen et al., 2024). The bone dielectric properties, i.e., its capacity to undergo electric polarization, resulting from charge displacement and the formation of dipoles, arise from the separation of hydrogen bonds present in its main components, collagen and hydroxyapatite (Jacob et al., 2018; Amin et al., 2019). The dielectric constant of bone tissue depends strongly on the water content and frequency of the applied electric field, with a value of 10 being reported for hydrated bone within the frequency range of 1–100 kHz (Bai et al., 2024). Previous studies have demonstrated a correlation between the dielectric coefficient of bone tissue and its elastic modulus and mineral density, suggesting that the mechanical performance and health condition of bone can be evaluated through the assessment of its dielectric properties (Sierpowska et al., 2003). Moreover, as a result of the bone tissue’s high degree of anisotropy, its conductivity value is highly dependent on the direction of the electrical flow through the tissue (Chen et al., 2024).

The primary origin of bone piezoelectricity arises from the non-centrosymmetric structure of COL1 fibers that, upon mechanical stimulation, slide over each other, creating a separation and polarization of charged groups, subsequently generating a physiologic electric potential (piezoelectric effect) (Barbosa et al., 2022; Bai et al., 2024). The piezoelectric coefficient of bone tissue has been reported to lie within the range of 0.7–2.3 pC/N, however, due to the tissue’s inherent anisotropy, these values vary across different regions (Chen et al., 2024). Importantly, the piezoelectric effect of bone tissue has been shown to induce polarization by converting mechanical loads in electrical signals, which, in turn, supports osteogenesis, bone growth, maintenance and healing (Rajabi et al., 2015). Accordingly, bone tissue has the capacity of regulating its metabolism and function through the conversion of mechanical loads into electrical signals, triggering several signaling cascades promoting cell osteogenic differentiation, tissue formation, and bone remodeling (Barbosa et al., 2022; Chen et al., 2024).

### 2.2 Articular cartilage

Articular cartilage is a highly specialized and complex connective tissue consisting in chondrocytes embedded in a dense ECM mainly composed by collagen type II (COL2) fibrils intertwined with proteoglycans—i.e., a core protein with covalently attached glycosaminoglycans (GAGs) (Sophia Fox et al., 2009). The cartilage tissue exhibits a multilayered gradient structure, where each distinct zone—superficial, middle, and deep—possesses unique characteristics tailored to its functional role (Gu et al., 2023). The superficial zone contains a high density of flattened chondrocytes, high water content, and an ECM rich in COL2 fibers arranged parallel to the surface, resulting in a smooth surface for reducing friction and high tensile strength to withstand shear forces. The middle zone contains rounded chondrocytes that are more sparsely distributed. Its ECM has a lower collagen concentration, with fibers that are organized in a more random orientation, and a higher proportion of proteoglycans, contributing to its ability to absorb compressive forces. Finally, the deep zone is characterized by the lowest chondrocyte density and water concentration. Here, the ECM is dominated by vertically aligned collagen fibers and a high concentration of proteoglycans, responsible for the tissue’s resistance to compressive loads.

Articular cartilage tissue has been shown to regulate its own metabolism through physical phenomena, namely, through physical interactions (electrical or mechanical signals) between the resident cells (chondrocytes and cartilage progenitor cells) and the surrounding ECM (Guilak et al., 2009). Such physical signals can induce and act synergistically with biochemical signals to modulate cartilage tissue formation and maintenance (Mow et al., 1999). In fact, such observations have motivated the combination of dynamic mechanical loading with chondrogenic growth factors (e.g., TGF-β) to promote cartilage ECM biosynthesis towards improved tissue engineering strategies (Mauck et al., 2003).

The electrical properties of the cartilage tissue arise from the flow of free cations—such as K+, Ca2+, and Na+—interacting with the fixed negative charges present on the carboxyl and sulfate groups attached to the main cartilage GAGs (i.e., chondroitin sulfate and hyaluronic acid). This interaction creates a dynamic environment where ions flow and diffuse, generating electrical phenomena such as diffusion and Donnan potentials (Farooqi et al., 2019; Miguel et al., 2022). Due to the high presence of COL2 fibers in its composition, cartilage also exhibits piezoelectric features, with a reported piezoelectric charge coefficient between 0.2 and 0.7 pC/N (Kapat et al., 2020). In comparison to bone, cartilage has a lower piezoelectric coefficient, possibly due to differences in the structure of the different collagen types as well as in the tissues’ dielectric properties (Denning et al., 2014). Notably, as in bone, the piezoelectricity of cartilage is crucial for the tissue’s maintenance and function. When cartilage is exposed to mechanical stress, the endogenous electrical signals generated by the COL2 fibers trigger key processes such as cell growth, differentiation and ECM production, thus promoting the tissue’s regeneration and mechanical performance (Poillot et al., 2021; Barbosa et al., 2022).

## 3 PEMF stimulators

**FIGURE 1:**
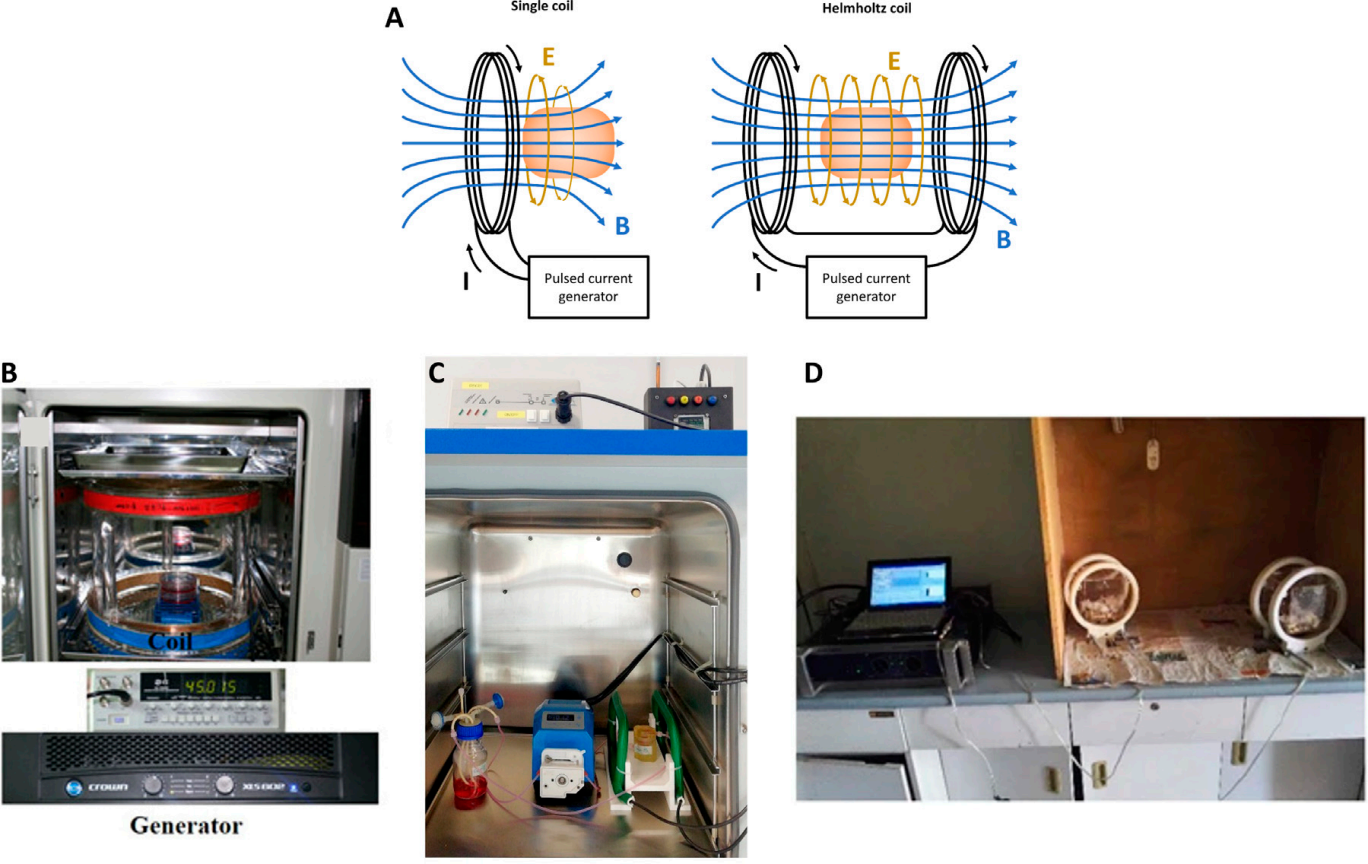
**(A)** Schematic representation of a general PEMF stimulator, composed of a single coil or a pair of Helmholtz’s coils; **(B)** Example of PEMF stimulation of 2D *in vitro* culture with custom-made device. Reproduced with permission from Kim et al, J Orthop Res. 39,8 (2021). Copyright 2020 Authors, licensed under a Creative Commons Attribution (CC BY) license; **(C)** Example of PEMF stimulation of 3D *in vitro* culture with IGEA BIOSTIM device. Reproduced with permission from Gabetti et al., Sci. Rep. 12, 13,859 (2022). Copyright 2022 Authors, licensed under a Creative Commons Attribution (CC BY) license; **(D)** Example of PEMF stimulation of *in vivo* culture with custom-made device. Reproduced with permission from Lei et al., Sci. Rep. 7,1 553 (2017). Copyright 2017 Authors, licensed under a Creative Commons Attribution (CC BY) license.

Several PEMF stimulators, both commercially available and custom-made, were used in the reported studies. In general, a PEMF device consists of a power supply connected to a single solenoid or a pair of Helmholtz’s coils (Figure 1A). In Figures 1B–D are reported examples of PEMF systems for stimulating 2D and 3D cell culture and animal models, respectively. According to Ampere’s law, these coils generate a time-varying electromagnetic field when powered with alternating current. By controlling the current that flows through the coils, a variety of PEMF stimulation waveforms can be generated, including triangular, square, sinusoidal, and trapezoidal waveforms (Hu et al., 2020). Just few PEMF stimulators are tunable in terms of magnetic field intensity (*B*), frequency (*f*), duty cycle (*DC*), pulse time (*t*), and/or waveform. In particular, the majority of the commercial devices (listed in Table 2) deliver a triangular or quasi-triangular waveform, with magnetic field intensity and frequency values varying, depending on the device, from 1.5 to 12 mT and from 8 Hz to 50 kHz, respectively. Some devices, such as the OrthoPulse (OSSATEC Benlux, Netherlands) and a stimulator by Tianjin Tongye (China), do not provide information about the waveform. It is noteworthy that the Biomet EBI Bone Healing System (Zimmer Biomet, United States), which was the first device obtaining the FDA pre-market approval in 1979 (at the time under the name Bio Osteogen System 204) (Orthopaedic and Rehabilitation Devices Panel, 2020), employs the sinusoidal waveform. The heterogeneity among available PEMF stimulators complicates direct comparisons of outcomes, making challenging to draw definitive conclusions or to establish consistent correlations between specific PEMF settings and their biological effects across different studies.

**TABLE 2:** Commercially available PEMF stimulators and operating parameters.

Device’s name	Company, country	Waveform	Magnetic field intensity (*B*)	Frequency (*f*)	Pulse time (*t*)	Tunability	References of studies using the stimulator
BIOSTIM	IGEA, Italy	quasi-triangular	2.0 mT ± 0.5 mT	75 Hz	1.3 ms	-	De Mattei et al. (2001), 2020; Nicolin et al. (2007), Saino et al. (2011), Esposito et al. (2013), Veronesi et al. (2015), Bagheri et al. (2018), Martini et al. (2020), Stefani et al. (2020), Gabetti et al. (2022), Daou et al. (2024), Scocozza et al. (2024)
I-ONE	IGEA, Italy	quasi-triangular	2 mT	75 Hz	-	-	Ongaro et al. (2012), Veronesi et al. (2015)
SpinalStim	Orthofix, United States	triangular	up to 3 mT	1–50 kHz	-	*B*, *f*	Selvamurugan et al. (2017), He et al. (2018)
PhysioStim	Orthofix, United States	triangular	up to 9 mT	1–50 kHz	-	*B*, *f*	Chen et al. (2013)
XT-2000B	Tianjin xtmed Co., China	triangular	3.8 mT	8 Hz	0.2 ms	-	Zhou et al. (2013)
	Hunan Forever Elegance Technology Co., Ltd., China	triangular	8 mT	20 Hz	-	-	Zhou et al. (2017)
Osteoplus	Fisiokinetec, Italy	square or triangular	up to 12 mT	15–120 Hz	DC = 20% or 50%	*B*, *f*, *DC*, waveform	Esposito et al. (2013)
BioMedsa	SDU Teknokent, Turkey	square	0.8 mT	7.3 Hz	-	-	Topal et al. (2020)
BTL-4000	BTL, United States	square	1–10 mT	15 Hz	-	*B*	Liu et al. (2021)
Fisiofield Mini	Fisioline Co., Italy	square	0–10 mT	10–100 Hz	5–50 ms	*f*, *t*	Reihani Kermani et al. (2014)
Biomet^®^ EBI Bone Healing System	Zimmer Biomet, United States	trapezoidal	1.8 mT	15 Hz	4.5 ms bursts of pulses, 225 μs each pulse	-	Murray and Pethica (2016)
OrthoPulse	OSSATEC Benlux BV, Netherlands	-	0.1 mT	15 Hz	5 ms	-	Kaivosoja et al. (2012), van der Jagt et al. (2012)
	Tianjin Tongye Science and Technology Co., China	-	8–10 mT	20 Hz	-	-	Bao et al. (2019)

## 4 Quantitative descriptor for comparing PEMF stimulation protocols

To address the challenge of comparing PEMF stimulation protocols and biological outcomes obtained using different PEMF stimulators and applying different operating parameters, we propose the use of a dedicated quantitative descriptor. The specifically developed descriptor is the PEMF dose, a comprehensive metric that integrates the magnetic field intensity, the stimulation waveform, and the exposure duration to the magnetic field. In detail, we calculated the PEMF dose (*D*), expressed in mT⋅h, as in Equation 1:

$$D=B_{rms}\;\cdot \;t_{\exp}$$ (1)

where *B*_*rms*_ is the root mean square value of the magnetic field intensity (in mT), and *t*_*exp*_ is the total exposure duration (in h) to the PEMF stimulation. For the different stimulation waveforms, *B*_*rms*_ was calculated from the peak amplitude of the magnetic field intensity (*B*_*peak*_) and the duty-cycle (*DC*), i.e., the fraction over one period of stimulation during which the stimulation is active, as shown in Table 3. Due to the variety of stimulating setups, for each study the *DC* was specifically calculated based on the reported PEMF waveform parameters.

**TABLE 3:** Formulas adopted for the calculation of B_rms_ depending on the waveform. B_peak_ is the peak intensity of the PEMF waveform, DC is the waveform duty cycle.

PEMF waveform	B_rms_
Square	$B_{rms}=B_{peak}\cdot \sqrt{DC}$
Triangular	$B_{rms}=\frac{B_{peak}}{\sqrt{3}}\cdot \sqrt{DC}$
Sinusoidal	$B_{rms}=\frac{B_{peak}}{\sqrt{2}}\cdot \sqrt{DC}$
Sawtooth	$B_{rms}=\frac{B_{peak}}{\sqrt{3}}\cdot \sqrt{DC}$
Offset sinusoid	$B_{rms}=B_{peak}\sqrt{\frac{DC}{2}+1}$

The *t*_*exp*_ was calculated as in Equation 2:

$$t_{\exp}={ED}_{day}\;\cdot \;{ED}_{week}\;\cdot \;n_{week},$$ (2)

where *ED*_*day*_ is the daily exposure duration to the PEMF stimulation (h/day), *ED*_*week*_ is the number of days of the week in which PEMF stimulation is applied (day/week), and *n*_*week*_ is the duration of the experiment expressed in weeks.

In Sections 5, 6, the PEMF dose has been calculated only for the studies that provided all the parameters needed for the evaluation (*B*_*peak*_, waveform, *DC*, *t*_*exp*_) while excluding the studies that did not provide information on the complete parameter set. The results are presented in *B*_*rms*_–*D* graphs. This visualization enables to appreciate the various PEMF stimulation protocols adopted, highlighting the contribution of the combined PEMF parameters (*B*_*rms*_) with respect to the total delivered stimulation (*D*). This method enabled a quantitative comparison of the effects of PEMF stimulation on bone and cartilage tissues for both *in vitro* and *in vivo* studies.

**FIGURE 2:**
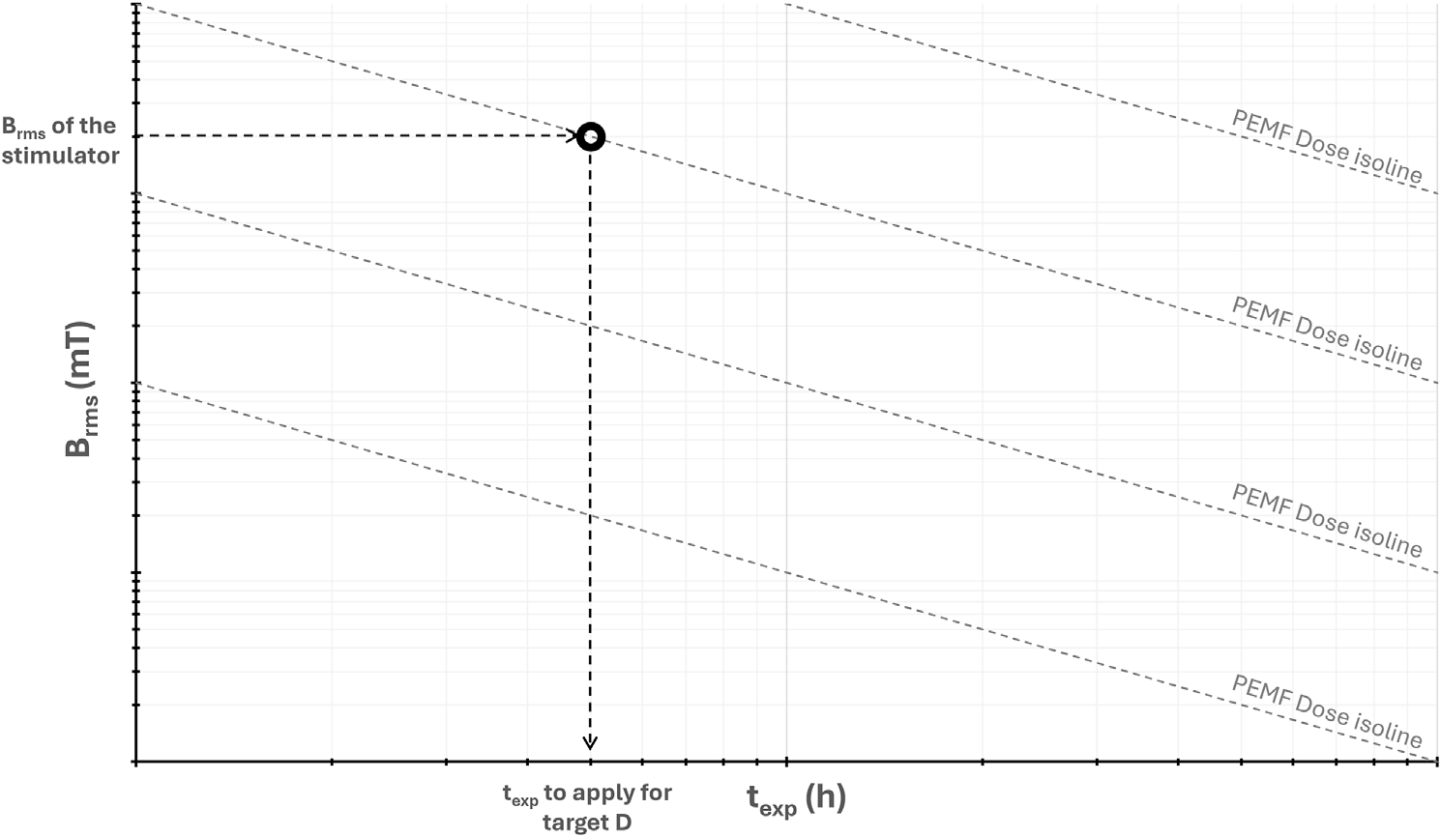
Log-log graph of the root mean square value of the magnetic field intensity (*B*_*rms*_) versus the total exposure duration (*t*_*exp*_), showing isolines of constant PEMF dose (*D*) and the procedure for extracting the value of *t*_*exp*_ for achieving the desired *D* value.

In addition, for visually representing and using the PEMF dose descriptor, we propose a log-log graph of *B*_*rms*_ versus *t*_*exp*_ *,* where isolines of constant *D* values appear as straight lines (Figure 2). Since the operating parameters of PEMF stimulators typically fall within a specific range of *B*_*rms*_ values, this graph enables the extraction of the corresponding *t*_*exp*_ value needed to achieve a target *D* value. Practically, starting from the *B*_*rms*_ value of the selected PEMF stimulator and tracing a horizontal line to intersect the desired D isoline, the corresponding t_exp_ value is extracted by drawing a vertical line downward to the *t*_*exp*_–axis, yielding the exposure duration required to deliver the target dose (Figure 2).

## 5 *In vitro* PEMF studies

Over the past few decades, numerous *in vitro* experiments have been conducted on bone and cartilage cells, as well as on 3D tissue models, to investigate the effects of PEMF stimulation and the associated activation of signaling pathways. In the following sections, these studies are presented based on the specific type of tissue stimulated.

### 5.1 Bone

For investigating the effects of PEMF stimulation on bone stem cells, differentiated cells, or even 3D constructs, the literature reports a wide range of values for each PEMF stimulation parameter. In particular, magnetic field intensity (*B*) values ranging from 0.1 to 20 mT and frequency (*f*) values ranging from 0.2 to 150 Hz were used, with variations in pulse time (*t*), duty cycle (*DC*), and waveform types (see Table 4).

**TABLE 4:** *In vitro* studies on the effects of PEMF stimulation on bone tissue.

Reference	Cell origin and type	PEMF stimulator	PEMF waveform	PEMF parameters	PEMF exposure duration	Main biological outcomes/Signaling pathways involved
Ongaro et al. (2014)	hBMMSCs and hASCs	BIOSTIM (IGEA, Italy)	quasi-triangular	*B* = 1.5 mT, *f* = 75 Hz; *t* = 1.3 ms	24 h/day for 28 days	hBMMSCs: ↑ ALP (until day 21); → ALP (from day 21) hASC: ↑ ALP (until day 21); ↓ ALP (from day 21)
Bagheri et al. (2018)	hBMMSCs	BIOSTIM (IGEA, Italy)	quasi-triangular	*B* = 1.5 mT; *f* = 75 Hz; *t* = 1.3 ms	24 h/day for 28 days	↑ Osteogenic differentiation activating the Notch canonical pathway
De Mattei et al. (2020)	hBMMSCs	BIOSTIM (IGEA, Italy)	quasi-triangular	*B* = 1.5 mT; *f* = 75 Hz; *t* = 1.3 ms	24 h/day for 28 days	↑ miRNAs expression
Martini et al. (2020)	hBMMSCs	BIOSTIM (IGEA, Italy)	quasi-triangular	*B* = 1.5 mT; *f* = 75 Hz; *t* = 1.3 ms	24 h/day for 28 days	↑ RUNX-2, ALP (at day 14), OCN (at day 28), p38 MAPK phosphorylation, SMAD activation
Gabetti et al. (2022)	hBMMSCs	BIOSTIM (IGEA, Italy)	quasi-triangular	*B* = 1.5 mT; *f* = 75 Hz; *t* = 1.3 ms; cultured under bidirectional perfusion of 0.3 mL/min	24 h/day for 14 days	↑↑ COL1
Daou et al. (2024)	hBMMSCs	BIOSTIM (IGEA, Italy)	quasi-triangular	*B* = 1.5 mT; *f* = 75 Hz; *t* = 1.3 ms; cultured under bidirectional perfusion of 0.3 mL/min	4 h/day for 21 days	↑ Angiogenic effects ↑ Osteogenic effects: COL1; COL1/COL2 ratio
Wang et al. (2019a)	Rabbit BMMSCs	custom-made	sinusoidal	*B* = 1 mT; *f* = 15 Hz; cultured under perfusion of 10 mL/min	4 h/day for 14 days	↑ ALP (on day 7 and 14), COL1, RUNX-2, Wnt-1, Lrp6 and β-catenin (on day 7); → Proliferation
Bloise et al. (2018)	hBMMSCs	BIOSTIM (IGEA, Italy)	quasi-triangular	*B* = 2 mT; *f* = 75 Hz; *t* = 1.3 ms	10 min/day for 28 days	↑ Ca^++^ production, RUNX-2, COL1, FN, BOSP, Osterix, OSC, BMP-2, ALP
Saino et al. (2011)	hBMMSCs	BIOSTIM (IGEA, Italy)	quasi-triangular	*B* = 2 mT; *f* = 75 Hz; *t* = 1.3 ms	20 min/day for 28 days	↑ OCN, OP, COL1
Petecchia et al. (2015)	hBMMSCs	BIOSTIM (IGEA, Italy)	quasi-triangular	*B* = 2 mT; *f* = 75 Hz; *t* = 1.3 ms	10 min/day for 27 days	↑ Ca^2+^ current, OPN; → Viability, Calcium deposition, ALP, COL1
Scocozza et al. (2024)	hASCs	BIOSTIM (IGEA, Italy)	quasi-triangular	*B* = 2 mT; *f* = 75 Hz; *t* = 1.3 ms	24 h/day for 21 days	→ osteogenic differentiation with PEMF alone ↑ osteogenic differentiation when cells seeded on hydroxyapatite-laden scaffolds
Selvamurugan et al. (2017)	hBMMSCs	Spinal Stim (Orthofix, United States)	triangular	*B* = 3 mT; *f* = 15 Hz	4 h/day for 33 days	↑ proliferation, differentiation activating TGF-β pathway; ↓ Smad7
He et al. (2018)	hBMMSCs	custom-made	triangular	*B* = 3 mT; *f* = 15 Hz	4 h/day for 9 days	↓ Osteoclastic differentiation; ↑ OPG
Zhou et al. (2011)	Rat osteoblast progenitor cells	custom-made	sinusoidal	*B* = from 0.9 to 4.8 mT; *f* = 50 Hz	30 min/day for 15 days	0.9–1.8 mT and 3.0–3.6 mT: ↑ ALP, differentiation and mineralization (at day 9); ↓ proliferation
Yong et al. (2014)	Rat BMMSCs	custom-made	sinusoidal	*B* = 1 mT; *f* = 15 Hz	4 h/day for 9 days	↑ Osteogenic differentiation; activation of PKA, ERK1/2 pathways
Kaivosoja et al. (2012)	- Saos 2 - hBMMSCs	Bone growth stimulator (OSSATEC Benlux BV, Netherlands)	-	*B* = 0.1 mT; *f* = 15 Hz; *t* = 5 ms	24 h/day for 28 days	↑ Viability, ALP (at day 21) and COL1 (at day 14)
Ehnert et al. (2018)	- Co-culture with human osteoblasts and hASCs - Osteoclasts	Somagen device	triangular	*B* = 0.282 mT; *f* = 16 or 26 Hz	- hOb + hASC: 7 min/day, 5 days/week for 2 weeks - Oc: 7 min/day for 5 days	↑ mitochondrial activity, ALP and matrix mineralization
Miyamoto et al. (2019)	MC3T3-E1	custom-made	sinusoidal	*B* = 0.1 and 0.4 mT; *f* = 10 Hz	1) 8 h/day for 21 days 2) 10 min of stimulation +20 min of rest for 3 times per day for 21 days	1) → ALP 2) ↑ Proliferation; → ALP
Suryani et al. (2019)	MC3T3-E1	custom-made	sinusoidal	*B* = 0.6 mT; *f* = 50 Hz; *t* = 3 ms	15, 30 or 60 min/day for 28 days	↑ Proliferation (15 min/day); → viability, osteogenic differentiation
Wang et al. (2019b)	Rat osteoblasts	custom-made	square	*B* = 0.6 mT; *f* = 50 Hz, DC = 50%	- ST: 15, 30, 60, 90 and 120 min - LT: 90 min/day for 12 days	- ST: ↑ ALP, RUNX2, OSX, BMP2 (after 30 min); activation of sAC-cAMP-PKA-CREB pathway (after 15 min) - LT: ↑ RUNX2, OSX and BMP2
Chen et al. (2023)	SCP-1	Somagen device	triangular	*B* = 0.282 mT; *f* = 16 Hz	- Continuous: 30 min every 24 h for 28 days - Intermittent: 10 min every 8 h for 28 days	Effects more pronounced in the intermittent group than in continuous one ↑↑ ALP, Piezo1 expression ↑↑ Ca^2+^ influx
Luo et al. (2012)	hBMMSCs	custom-made	-	*B* = 1.1 mT; *f* = from 5 to 150 Hz	30 min/day for 21 days	↑ ALP (at day 3, higher at 50 Hz), OCN (at day 21, higher at 50 Hz)
Yin et al. (2018)	hASCs	custom-made	-	*B* = 1 mT; *f* = 50 Hz	2 h/day for 21 days	↑ Proliferation (until day 7), ALP (at day 7), OPN (at day 21) and OCN (at day 21); ↓ Proliferation (from day 7 to day 21)
Pi et al. (2019)	RAW264.7	custom-made	sinusoidal	*B* = 1 mT; *f* = 75 Hz	3 h/day for 4 days	↓ Osteoclastic differentiation, ROS; → Viability
(Kim et al., 2021, p. 202)	Saos-2	custom-made	sinusoidal	*B* = 1 mT; *f* = 45 Hz	8 h/day for 7 days	↑ COL1, OCN, BMP2, OP, OPG, pERK, p38; → Proliferation
Zhou et al. (2014)	Rat osteoblast progenitor cells	custom-made	1) sinusoidal 2) triangular 3) square 4) sawtooth	*B*_*rms*_ = 1.8 mT; *f* = 50 Hz	30 min/day for 12 days	1) ↑ OB differentiation; ↓ proliferation 2) ↑ OB differentiation 3) ↑ OB proliferation; → differentiation 4) → differentiation

Several researchers adopted a specific PEMF parameter combination (*B* = 1.5 mT, *f* = 75 Hz, *t* = 1.3 ms, and a quasi-triangular waveform), which can be obtained using the commercially available PEMF stimulator BIOSTIM (IGEA, Italy). These studies demonstrated that exposing hBMMSCs to this PEMF protocol effectively promoted their commitment to the osteogenic lineage (Ongaro et al., 2014; Bagheri et al., 2018; De Mattei et al., 2020; Martini et al., 2020; Gabetti et al., 2022; Daou et al., 2024). Adopting the same protocol, monolayers of hBMMSCs and human adipose-derived stem/stromal cells (hASCs) exposed to PEMF for 24 h/day over 28 days resulted in an increase in alkaline phosphatase (ALP) expression until day 21. Subsequently, ALP levels remained stable in hBMMSCs but decreased in hASCs (Ongaro et al., 2014). Martini and colleagues later demonstrated that ALP expression peaked at day 14, accompanied by increased expression of runt-related transcription factor 2 (RUNX2) and osteocalcin (OCN) genes. In addition, the same PEMF protocol was shown to activate the bone morphogenetic protein (BMP) signaling pathway, leading to increased expression of the receptors ALK2, phosphorylation of p38 mitogen-activated protein kinases (MAPK), and activation of Smad 1/5/8, a transcription factor closely associated with the BMP2 pathway (Martini et al., 2020). In addition, it was demonstrated that this PEMF parameter combination applied on hBMMSCs modulated the Notch genes involved in osteogenic differentiation, and increased microRNA and the vascular endothelial growth factor (VEGF) expression, which are both essential for promoting osteogenesis and angiogenesis (Bagheri et al., 2018; De Mattei et al., 2020). To explore the effects of PEMF stimulation on a more realistic model, some of the authors used the same PEMF protocol, (for 24 h/day over 14 days) for stimulating a 3D construct consisting of a porous scaffold seeded with hBMMSCs and cultured within a perfusion bioreactor. The combination of PEMF stimulation and bi-directional direct perfusion, this latter providing physiological shear stress stimuli, resulted in a synergistic effect, with enhanced expression of COL1, ALP, and RUNX2 (Gabetti et al., 2022), in accordance with (Wang H. et al., 2019). Using the same bioreactor and PEMF stimulation set-up, Daou and colleagues demonstrated through transcriptomics analysis that PEMF stimulation promoted the expressions of angiogenesis and osteogenesis upstream regulators and activated immune response pathways, effectively replicating *in vitro* the dynamic interplay of biological processes developing during bone healing (Daou et al., 2024). Imposing the same frequency, pulse time, and waveform, but increasing the magnetic field intensity (2 mT), comparable effects on hBMMSCs were observed. In particular, calcium production and the expression of genes such as OCN, osteopontin (OP), COL1, RUNX2, fibronectin (FN), bone sialoprotein (BOSP), osterix (OSX), BMP2, and ALP increased after 28 days of PEMF stimulation for either 10 min/day (Bloise et al., 2018) or 20 min/day (Saino et al., 2011). Interestingly, despite using the same PEMF protocol, two studies reported no changes in calcium deposition, ALP, or COL1 expression (Petecchia et al., 2015; Scocozza et al., 2024).

Besides the differentiation of hBMMSCs toward osteoblasts, it was also demonstrated that imposing a specific PEMF stimulation (*B* = 3 mT, *f* = 15 Hz, triangular waveform) may reduce osteoclastic differentiation (Selvamurugan et al., 2017; He et al., 2018). Optimal ALP expression and mineralization were also observed in rat osteoblast progenitor cells imposing *B* = 3.0–3.6 mT (Zhou et al., 2011). Although characterized by a lower magnetic field intensity, the PEMF stimulation protocol (*B* = 1 mT, *f* = 15 Hz, sinusoidal waveform, for 4 h/day) applied on rat BMMSCs activated, after 9 days, osteogenic pathways, including MEK/ERK and PKA-ERK1/2 pathways (Song et al., 2014; Yong et al., 2014). For lower magnetic field intensity values (0.1–0.6 mT), there is no agreement regarding the efficacy for osteogenic differentiation (Kaivosoja et al., 2012; Ehnert et al., 2018; Miyamoto et al., 2019; Suryani et al., 2019; Wang Y. et al., 2019; Chen et al., 2023). Concerning the frequency parameter, the cell osteogenic differentiation has been promoted when compared to a non-stimulated control when similar PEMF intensities (1–1.1 mT) were applied, despite different frequency values (45, 50, or 75 Hz) (Luo et al., 2012; Yin et al., 2018; Pi et al., 2019; Kim et al., 2021).

Interestingly, Zhou and colleagues demonstrated that cell proliferation can be affected by PEMF stimulation by varying the waveform type. Imposing a specific PEMF stimulation protocol (*B*_*rms*_ = 1.8 mT, *f* = 50 Hz) with a sinusoidal waveform, the proliferation of rat osteoblast progenitor cells was observed to decrease in favor of differentiation, while using a square waveform the cell proliferation was enhanced (Zhou et al., 2014). These findings were partially confirmed by two further studies, where PEMF stimulation at *B* = 1 mT with sinusoidal waveforms at different frequencies (*f* = 15 and 45 Hz, respectively) were applied, without significant changes in proliferation rates during the experimental time (Wang H. et al., 2019; Kim et al., 2021). Additionally, a triangular waveform has been shown to promote the proliferation of bone progenitor cells (Selvamurugan et al., 2017; Miyamoto et al., 2019).

In summary, although the high variability in operating parameters, it is interesting to note that PEMF stimulation applied on bone stem cells generally induces osteogenic differentiation, which is associated with the activation of various signaling pathways. These pathways are known to be closely linked with the expression of specific osteogenic markers. In detail, the reviewed studies indicate that PEMF affects adenosine receptors and calcium channels which play a key role in the activation and mediation of the downstream osteogenic effects. In fact, PEMF stimulation enhances the expression and activity of adenosine A2A/A3 receptors on MSCs and osteoblasts, which are key resident cells involved in bone remodeling. The activation of A2A/A3 receptors triggers the sAC-cAMP-PKA-CREB pathway, which promotes the expression of the osteogenic transcription factors osterix (OSX) and RUNX2, which in turn enhance the synthesis of bone ECM proteins (e.g., osteocalcin and collagen type I) while suppressing the secretion of inflammatory cytokines, both favoring bone formation (Kar et al., 2021). In addition, PEMF enhances the levels of intracellular calcium ions by the modulation of two types of calcium channels, voltage-gated calcium channels (VGCCs) and store-operated calcium channels (SOCs). Then, the elevated calcium levels trigger both the MAPK/ERK, Wnt/β-catenin, and Calmodulin/CaMK pathways, all of which are known to enhance ALP activity, osteoblast proliferation, MSC osteogenic differentiation and ECM mineralization (Gavazzo et al., 2021). Furthermore, as also recently reported by Kaadan and colleagues (Kaadan et al., 2024) PEMF stimulation activates the Notch canonical, MAPK-ERK1/2, Wnt, mTOR, and sAC-cAMP-PKA-CREB pathways (Supplementary Table S1), which play a pivotal role in bone formation. The synthesis of ECM proteins is also enhanced by various activated growth factors, including IGF and TGF-β. Concurrently, PEMF stimulation inhibits osteoclastogenesis initiated by RANK pathways, thereby reducing the rate of bone resorption.

### 5.2 Quantitative comparison among *in vitro* studies on PEMF stimulation for bone tissue

**FIGURE 3:**
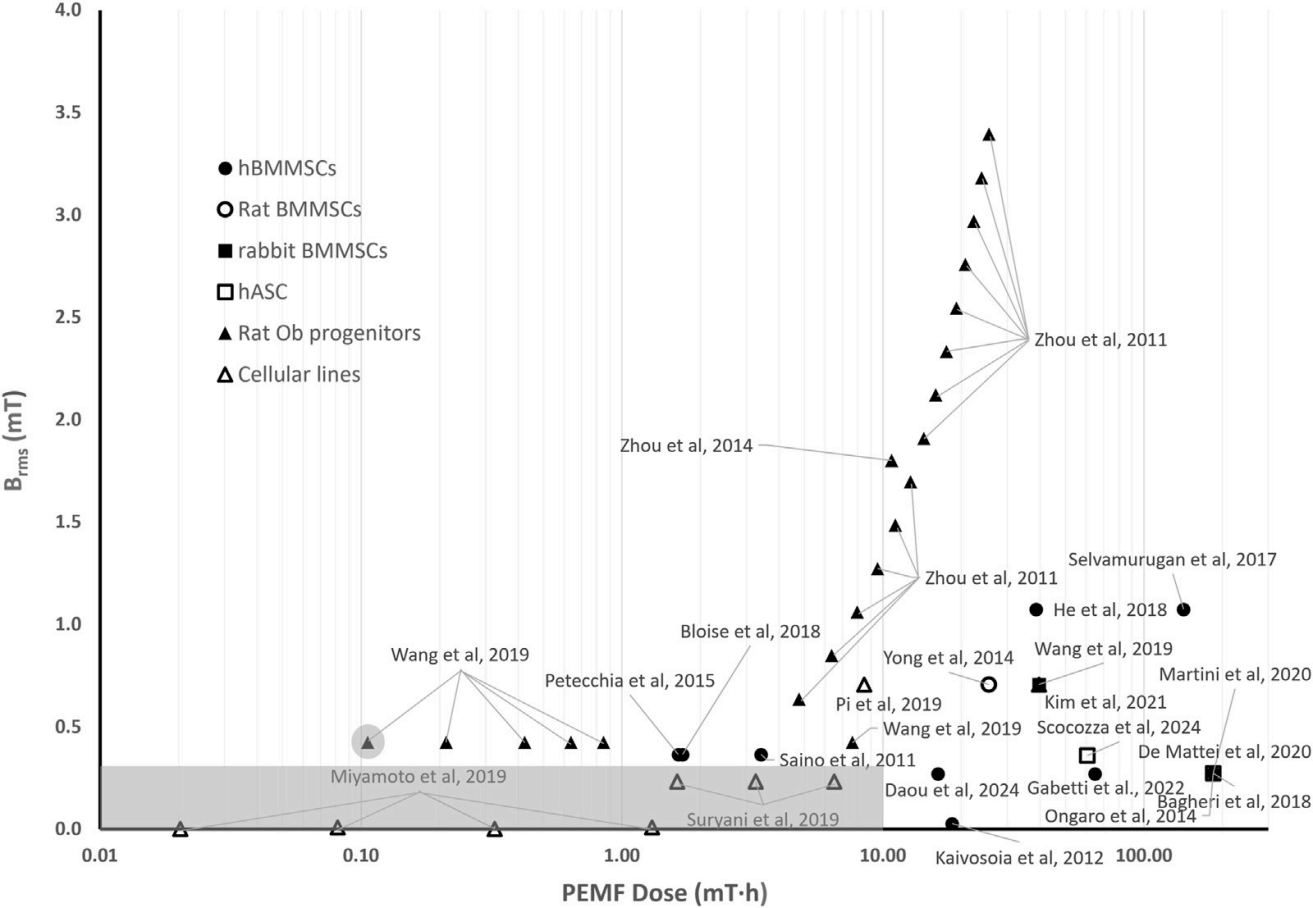
Distribution of the root mean square value of the magnetic field intensity (*B*_*rms*_) and the PEMF dose (*D*) value for the *in vitro* studies performed imposing PEMF stimulation on bone cells/tissues. The grey rectangle represents the threshold region for the *B*_*rms*_ and *D* values, within which the likelihood of observing an osteogenic effect induced by PEMF stimulation is lower. The different symbols refer to different cell types.

Figure 3 shows the distribution of the root mean square values of the magnetic field intensity (*B*_*rms*_) and the PEMF dose (*D*) values (in logarithmic scale) for some of the *in vitro* studies performed imposing PEMF stimulation on bone cells or constructs and described in the previous section. The distribution reflects the wide range of parameters and exposure durations that were adopted, resulting in PEMF dose values ranging from 10^–2^ to 2⋅10^2^ mT·h.

Interestingly, in studies where the applied *B*_*rms*_ was below 0.3 mT and the resulting *D* value was less than 10 mT∙h (grey rectangle in Figure 3), a delay in ALP expression or even no ALP expression was observed. In detail, Miyamoto and colleagues, who exposed osteoblastic MC3T3-E1 cells to *B*_*rms*_ values equal to 0.002 mT and 0.008 mT (*f* = 10 Hz) with *D* values ranging from 0.02 to 1.3 mT·h (Miyamoto et al., 2019), did not register any increase in ALP expression applying either a short term (30 min/day) or a long-term (8 h/day) exposure. Accordingly, Suryani et al., who applied to MC3T3-E1 cells a *B*_*rms*_ value equal to 0.232 mT (*f* = 50 Hz) associated with PEMF dose values (*D* = 1.6, 3.2, 6.5 mT·h) lower than 10 mT∙h, did not observe increase in ALP expression for any of the applied exposure durations (Suryani et al., 2019). Overall, the results of different studies suggest that, when *B*_*rms*_ values lower than 0.3 mT are adopted, it is fundamental to increase the delivered PEMF dose above 10 mT∙h for observing osteogenic behaviors. In particular, an increase in osteogenic markers after 21 days of stimulation when hBMMSCs were exposed to low-intensity PEMFs (*B*_*rms*_ = 0.027 mT, *f* = 15 Hz) but with a total PEMF dose of 18.4 mT∙h was reported (Kaivosoja et al., 2012). Similarly, when a *B*_*rms*_ value of 0.27 mT (f = 75 Hz) was applied, enhanced osteogenic differentiation was observed in both static monolayer cultures of hBMMSCs or hASCs, characterized by a PEMF dose of 184 mT∙h (Ongaro et al., 2014; Bagheri et al., 2018; De Mattei et al., 2020; Martini et al., 2020), as well as in hBMMSCs-seeded scaffolds cultured under perfusion with PEMF doses of 64.9 mT∙h (Gabetti et al., 2022) and 16.3 mT∙h (Daou et al., 2024).

Remarkably, for *B*_*rms*_ values above 0.3 mT, intensity-dependent effects could not be observed, but from the literature studies analyzed it appears that a minimum value of PEMF dose should be delivered. Specifically, Wang and colleagues, who applied to rat osteoblasts a square PEMF waveform (*B* = 0.6 mT, *f* = 50 Hz, *DC* = 50%, varying the exposure from 15 to 120 min) resulting in *B*_*rms*_ = 0.424 mT, found that the group exposed to 15 min of PEMF (corresponding to *D* = 0.11 mT∙h) did not show any significant differences in comparison to the untreated group. Differently, the experimental groups subjected to longer exposure durations, thus receiving higher PEMF doses (with *D* ranging from 0.21 to 7.64 mT∙h), reported increased osteogenic differentiation (Wang Y. et al., 2019). This was confirmed by Zhou et al. that exposed rat osteoblastic progenitor cells to sinusoidal PEMFs of different intensities (*B* = 0.9–4.8 mT, *f* = 50 Hz, for 30 min/day for 15 days) (Zhou et al., 2011), corresponding to *B*_*rms*_ values ranging from 0.64 to 3.40 mT with PEMF dose values ranging from 4.77 to 25.46 mT∙h. All stimulation protocols promoted differentiation and mineralization of osteoblasts and increased osteogenesis-related gene expression (Zhou et al., 2011). Overall, in studies in which *B*_*rms*_ values above 0.3 mT and *D* values equal or above 0.21 mT∙h were applied, the authors reported increased osteogenic differentiation (Saino et al., 2011; Ongaro et al., 2014; Yong et al., 2014; Petecchia et al., 2015; Selvamurugan et al., 2017; Bagheri et al., 2018; Bloise et al., 2018; De Mattei et al., 2020; Martini et al., 2020; Kim et al., 2021; Scocozza et al., 2024) or decreased osteoclastic differentiation (He et al., 2018; Pi et al., 2019) on a wide variety of cell types (Figure 3).

To sum up, the proposed quantitative comparison made it possible to deduce that, for low *B*_*rms*_ values (<0.3 mT), the exposure duration plays a crucial role, as enhanced osteogenic differentiation was observed only when a PEMF dose above 10 mT∙h was reached. When higher magnetic field intensities (*B*_*rms*_ ≥ 0.3 mT) are provided, the exposure duration becomes less relevant, although a minimum PEMF dose threshold (*D* ≥ 0.21 mT∙h) is required to induce osteogenic effects.

### 5.3 Cartilage

PEMF stimulation has recently gained attention also as a promising non-invasive treatment for enhancing the clinical outcome of cartilage repair procedures, showing both anti-inflammatory and chondrogenic effects on chondrocyte cultures (Ongaro et al., 2012; Varani et al., 2021). Analogously to PEMF studies for bone tissue, the stimulation parameters applied for investigating the PEMF effects on cartilage cells encompass a wide range of values, from 1 to 4 mT for the magnetic field intensity and from 15 to 75 Hz for the frequency (see Table 5). Moreover, different culture techniques have been employed, including the conventional monolayer culture method, the cultivation of cells as 3D pellets for mimicking the native cell condensation during chondrogenensis, and 3D cartilage constructs based on cell-seeded scaffolds. In particular, the application of PEMF stimulation (*B* = 1.5 mT, *f* = 75 Hz, quasi-triangular waveform, for 24 h/day) to 3D pellets of MSCs derived from bovine synovial fluid and treated with interleukin (IL-1β) resulted in an increase in COL2 and aggrecan (ACAN) gene expression after 5 weeks of treatment. This demonstrated the promotion of the MSCs chondrogenic differentiation even under inflammatory conditions (Ongaro et al., 2012). Similarly, PEMF treatment (*B* = 2 mT, *f* = 75 Hz, quasi-triangular waveform, for 8 h/day) applied to human umbilical cord derived MSCs was observed to significantly enhance COL2 expression, with no differences between the devices utilized (Esposito et al., 2013). Differently, stimulating 3D pellets of rabbit BMMSCs with a similar protocol (i.e., *B* = 1.8 mT, *f* = 75 Hz for 8 h/day) resulted in no changes in COL2 expression and GAGs production (Kavand et al., 2019). Concerning hASCs, Chen et al. observed that exposing 3D pellets to PEMF stimulation (*B* = 2 mT, *f* = 15 Hz, triangular waveform, for 8 h/day) led to an increase in the expression of transcription factor SOX9, ACAN, COL1, and OCN after 5 days and enhanced COL2 expression after 7 days of culture (Chen et al., 2013). Even shorter exposure durations (i.e., 3 h/day or 10 min applied once with *B* = 1–4 mT and *f* = 15 Hz, square waveform) could be sufficient to enhance the expression of COL2, SOX9, and ACAN genes in human (Parate et al., 2017; 2020; Celik et al., 2021) and rat BMMSCs (Li et al., 2023). However, the application of PEMF stimulation (*B* = 2 mT, *f* = 15 Hz, sinusoidal waveform) to chondroprogenitors harvested from non-diseased human knee joints for 10 min every 3 days over a period of 21 days did not result in any significant increase in the expression of chondrogenic markers (Vinod et al., 2021). This discrepancy could be attributed to differences in experimental protocols, such as the cell type, the duration and frequency of stimulation, or the specific waveform adopted. Applying a different stimulation protocol (*B* = 1.6 mT, *f* = 75 Hz, sinusoidal waveform) for 4 h/day for 10 days to human matrix-induced autologous chondrocyte implantation (MACI)-derived cells, it was shown that cell proliferation could be increased until day 7, as well as the synthesis of COL2 (Nicolin et al., 2007). Similarly, De Mattei and colleagues demonstrated that for increasing the proliferation rate of human articular chondrocytes at least 9 h of PEMF stimulation (*B* = 2.3 mT, *f* = 75 Hz, quasi-triangular waveform) are necessary. Moreover, they observed that PEMF can induce cell proliferation of low density chondrocyte cultures for a long time (6 days) only when fresh serum is added again in the culture medium. In other words, the PEMF exposure seemed to accelerate the consumption of growth factors and therefore the proliferation rate (De Mattei et al., 2001). Finally, Hilz et al. demonstrated that exposing 3D cartilage constructs, based on polyurethane scaffolds seeded with articular chondrocytes from bovine fetlock joints, to combined PEMF stimulation (*B* = 1–3 mT, *f* = 60 Hz, sinusoidal waveform, for 3 h every 2 days) and mechanical stimulation led to higher levels of GAG content and COL2, cartilage oligomeric matrix protein (COMP), SOX9, proteoglycan-4 (PRG-4) and matrix metalloproteinase 3 and 13 (MMP-3 and 13) expressions in comparison to PEMF stimulation alone (Hilz et al., 2014).

**TABLE 5:** *In vitro* studies on the effects of PEMF stimulation on cartilage tissue.

Reference	Cell origin and type	PEMF stimulator	PEMF waveform	PEMF parameters	PEMF exposure duration	Main biological outcomes/Signaling pathways involved
Ongaro et al. (2012)	MSCs from bovine synovial fluid	I-ONE (IGEA, Italy)	quasi-triangular	*B* = 1.5 mT; *f* = 75 Hz; *t* = 1.3 ms	24 h/day for 3 and 5 weeks	→ GAG deposition, PG ↑ COL2, ACAN, differentiation in inflammatory conditions
Esposito et al. (2013)	MSCs from human umbilical cord	- BIOSTIM (IGEA, Italy) - Osteoplus, (Fisiokinetec, Italy)	- quasi-triangular - triangular	*B* = 2 mT; *f* = 75 Hz	8 h/day for 21 days	↑ ECM production (from day 14), COL2, GAGs production
Kavand et al. (2019)	Rabbit BMMSCs and chondrocytes	custom-made	-	*B* = 2.6 mT; *f* = 75 Hz, DC = 80%	8 h/day for 21 days	→ GAGs deposition, COL2
Chen et al. (2013)	hASCs	Physio Stim (Orthofix, United States)	triangular	*B* = 2 mT, *f* = 15 Hz; *t* = 5.46 ms	8 h/day for 10 days	↑ COL2 (at day 7) and, SOX9, ACAN, COL1 and OCN (at day 5); → Viability
Parate et al. (2017)	hBMMSCs	custom-made	square	*B* = 1, 2, 3, 4 mT; *f* = 15 Hz; *t* = 6 ms	5, 10, 20, 30 or 60 min once at the beginning of the differentiation period	2 mT, 10 min: ↑ COL2, SOX9 and ACAN
Parate et al. (2020)	hBMMSCs	custom-made	square	*B* = 1, 2, 3 and 4 mT; *f* = 15 Hz; *t* = 6 ms	−10 min once −30 min once (only with 2 mT)	2 mT, 10 min once: ↑ COL2, SOX9, ACAN
Celik et al. (2021)	hBMMSCs	custom-made	square	*B* = 1, 2 and 3 mT; *f* = 15 Hz; *t* = 6 ms	5, 10, 20 or 30 min for once, twice or trice	10 min once: ↑ SOX9, COL2, ACAN
Li et al. (2023)	Rat BMMSCs	Physio Stim (Orthofix, Texas, United States)	triangular	*B* = 2.5 mT; *f* = 15 Hz; *t* = 5.46 ms	3 h/day for 21 days	↑ SOX9, COL2, ACAN ↓ RUNX2; COL10 and MMP13
Vinod et al. (2021)	Human chondroprogenitors	custom-made	sinusoidal	*B* = 2 mT; *f* = 15 Hz; *t* = 6 ms	10 min every 3 days for 21 days	↑ GAGs deposition; → ACAN, SOX9, TGF-β1/2/3, COL1A1
Nicolin et al. (2007)	Human chondrocytes from 3D implanted scaffold	BIOSTIM (IGEA, Italy)	quasi-triangular	*B* = 1.6 mT; *f* = 75 Hz; *t* = 1.3 ms	- ST: 12 h for 2 days - LT: 4 h/day for 10 days	- ST: ↑ Proliferation (at day 2) - LT: ↑ Proliferation (at day 7 and 10), Chondroitin sulfate A and C synthesis, COL2
De Mattei et al. (2001)	Human chondrocytes	IGEA (Italy)	quasi-triangular	*B* = 2.3 mT; *f* = 75 Hz; *t* = 1.3 ms	- ST: from 1 to 18 h (once) - LT: 24 h/day for up to 6 days	- ST: ↑ Proliferation (after 9 h) - LT: ↑ H-thymidine incorporation (until day 3); ↓ H-thymidine incorporation (from day 5)
Hilz et al. (2014)	Calf chondrocytes	custom-made	sinusoidal	*B* = 1, 2 and 3 mT; *f* = 60 Hz Each condition applied in combination with mechanical stimulation (MEC)	3 h every 2 days for 21 days (not the same day as MEC)	- PEMF + MEC: ↑ COL2 and ACAN; ↓ COL1 - PEMF: ↑ homogeneous ECM production

Briefly, several studies demonstrated that PEMF may have beneficial influence for cartilage regeneration, particularly promoting the differentiation of stem cells towards chondrocytes, enhancing the expression of COL2, SOX9, ACAN genes, and increasing the production and deposition of GAGs, as well as the synthesis of cartilage-like ECM through adenosine receptors.

### 5.4 Quantitative comparison among *in vitro* studies on PEMF stimulation for cartilage tissue

**FIGURE 4:**
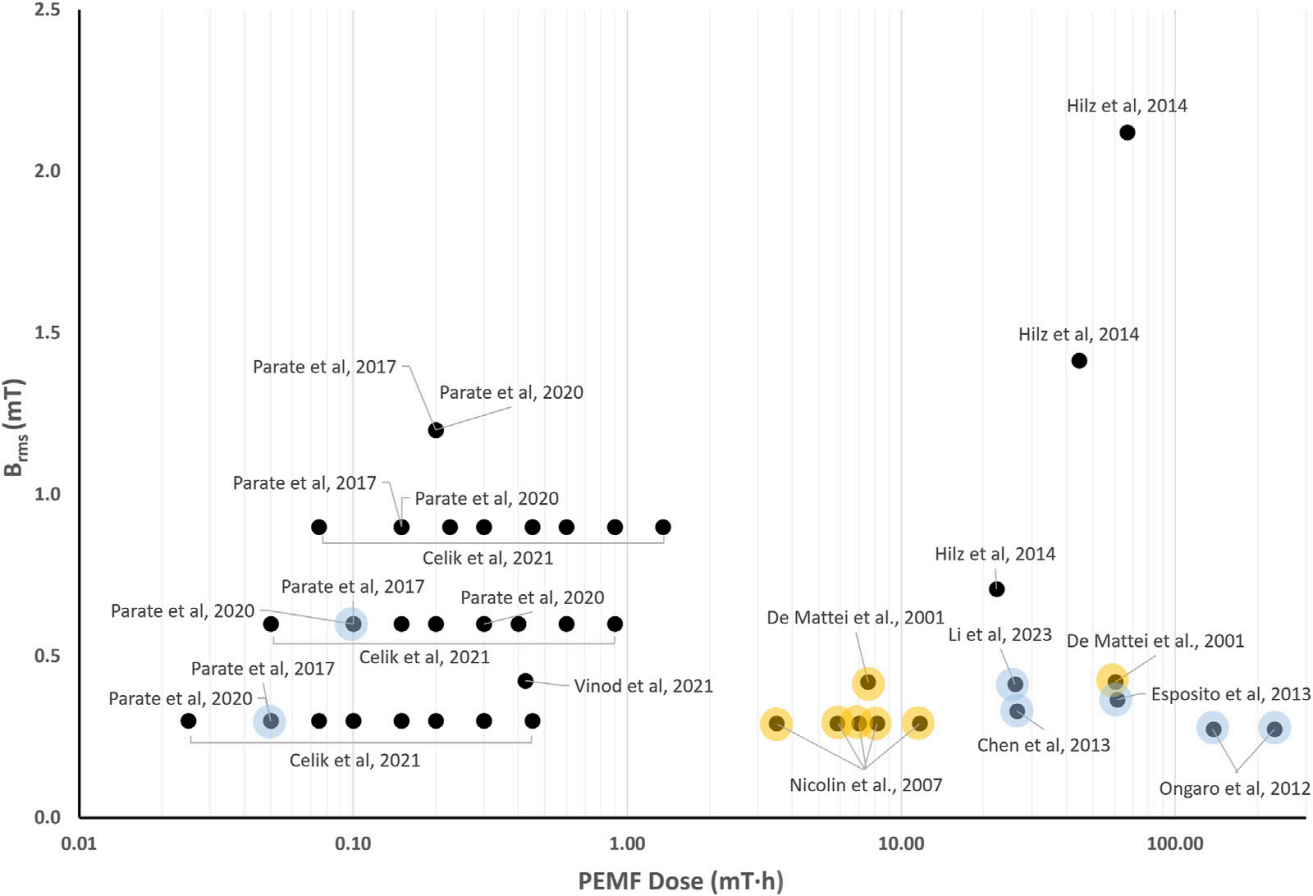
Distribution of the root mean square value of the magnetic field intensity (*B*_*rms*_) and the PEMF dose (*D*) value for the *in vitro* studies performed imposing PEMF stimulation on cartilage cells/tissues. The colored dots refer to studies reporting enhanced chondrocyte proliferation (yellow dots) or promoted chondrogenic differentiation (light blue dots).

Similarly to what was reported for the *in vitro* studies on bone cells, the wide PEMF parameter variation that characterize the *in vitro* studies for cartilage tissue results in *B*_*rms*_ values ranging from 0.25 to 2.1 mT and *D* values ranging from 2∙10^–2^ to 3∙10^2^ mT·h (Figure 4). Depending on the stimulated cells and on the combinations of *B*_*rms*_ and *D* values, different responses can be observed. In particular, the study conducted by De Mattei and co-workers, who subjected chondrocytes to PEMF (*B* = 2.3 mT, *f* = 75 Hz) resulting in *B*_*rms*_ value equal to 0.42 mT, led to chondrocyte proliferation imposing either 18 h (*D* = 7.56 mT∙h) or 6 days (*D* = 60.47 mT·h) of exposure duration (De Mattei et al., 2001). Increased chondrocyte proliferation was also observed when imposing a lower intensity PEMF stimulation (*B* = 1.6 mT, *f* = 75 Hz) (Nicolin et al., 2007), resulting in a *B*_*rms*_ value equal to 0.29 mT and *D* values ranging from 3.51 to 8.18 mT·h. These findings suggest that *B*_*rms*_ values equal to or greater than 0.3 mT associated with *D* values above 3.5 mT·h could be effective for inducing the proliferation of chondrocytes. Further studies exposing different types of stem cells to PEMF stimulation with *B*_*rms*_ values above 0.25 mT and *D* values higher than 26.02 mT∙h demonstrated increased chondrogenic differentiation (Ongaro et al., 2012; Chen et al., 2013; Esposito et al., 2013; Li et al., 2023).

In contrast, for *D* values below 3.5 mT∙h, no clear relationship between PEMF parameters and biological effects could be established. Recently, hBMMSCs were exposed to square PEMF waveform varying the intensity (*B* = 1–4 mT, *f* = 15 Hz) and the exposure duration (from 5 to 60 min). Chondrogenic differentiation was obtained only when *B* = 2 mT for 10 min was applied (Parate et al., 2017; 2020), corresponding to *B*_*rms*_ = 0.6 mT and *D* = 0.1 mT·h. However, in a subsequent study, the same combination applied to the same cell type proved ineffective, with chondrogenic differentiation observed only when *B* = 1 mT was imposed for 10 min (Celik et al., 2021), resulting in *B*_*rms*_ = 0.3 mT and *D* = 0.007 mT·h. In case of chondroprogenitors, exposed to a sinusoidal PEMF waveform (*B* = 2 mT, *f* = 15 Hz) resulting in *B*_*rms*_ = 0.424 mT and *D* = 0.424 mT·h, no effects on chondrogenic differentiation were detected (Vinod et al., 2021).

Overall, the quantitative comparison of *in vitro* studies applying PEMF stimulation to cartilage cells indicates that the exposure duration might be a crucial parameter, indeed increased chondrocyte proliferation was observed when applying *D* above 3.5 mT∙h and enhanced chondrogenic differentiation when applying *D* above 26.02 mT∙h. When lower PEMF doses were provided, the available studies reported different combinations of parameters for chondrogenic differentiation.

## 6 *In vivo* animal studies

The effects of PEMF stimulation on bone, cartilage, and osteochondral tissues have also been widely investigated *in vivo* through a considerable number of animal studies. The studies involved the use of a great variety of models, which differ for the investigated pathology (i.e., fracture healing, osteoporosis, bone resorption, and OA), for the selected animal (i.e., mice, rats, rabbits, guinea pigs, and dogs), and for the technique used to induce pathological conditions. Animals have been subjected to PEMF stimulation using either commercially available devices or custom-made setups, and, depending on the animal’s size, the stimulation was applied to a specific body district or to the whole body. The following sections recapitulate the main findings, depending on the specific type of tissue stimulated.

### 6.1 Bone

Analogously to *in vitro* studies, the *in vivo* investigation of the effects of PEMF stimulation on bone tissue also shows variations in the applied PEMF parameters among the studies, with magnetic field intensity ranging from 0.1 to 12.4 mT and frequency ranging from 1 to 100 Hz (see details in Table 6).

**TABLE 6:** *In vivo* studies on the effects of PEMF stimulation on bone tissue.

Reference	Species	Investigated condition	PEMF stimulator	PEMF waveform	PEMF parameters	PEMF exposure	Main biological outcomes/Signaling pathways involved
Bao et al. (2019)	rabbit	fracture healing	- Tianjin Tongye Science - Technology Co., China	-	*B* = 8–10 mT, *f* = 20 Hz	1 h/day, 5 weeks	↑ bone volume and trabecular structure
Liu et al. (2021)	rat	fracture healing	BTL-4000, BTL, United States	-	*B* = 1, 5, 10 mT, *f* = 15 Hz	2 h/day, 7 days	5, 10 mT: ↑ Ca, ALP in serum ↑ mineral density, maximum load, fracture load, elastic load, bending energy
Jing et al. (2016)	rabbit	implant integration	custom-made	square	*B* = 2 mT, *f* = 15 Hz, 5 ms bursts, *t* = 0.2 ms	2 h/day, 6–12 weeks	↑ osteogenesis ↑ Runx2, BMP2, OCN, Wnt/β-catenin expression
Cai et al. (2018)	rabbit	implant integration	custom-made	square	*B* = 2 mT, *f* = 15 Hz, 5 ms bursts, *t* = 0.2 ms	2 h/day, 8 weeks	↓ deterioration ↑ osteointegration ↑ Wnt/β-catenin signaling
Nunes et al. (2021)	rat	implant integration	custom-made	triangular	*B* = ± 1 mT, *f* = 15 Hz, 5 ms bursts, *t* = 0.2 ms	1, 3 h/day, 5 days/week, 6 weeks	1 h/day: ↑ removal torque, bone volume, mineral density, cell viability 3 h/day: ↑ trabecular bone thickness, cell proliferation
Barak et al. (2016)	rabbit	implant integration	custom-made	-	*B* = 0.2–0.4 mT, *f* = 10 Hz	2–4 weeks	↑ trabecular bone fraction ↑ trabecular number ↑ bone-to-implant contact
Androjna et al. (2014)	rat	osteoporotic fracture healing	custom-made	triangular	*B* = 0.5 mT, *f* = 15 Hz, 5 ms bursts, *t* = 0.26 ms	3 h/day, 6 weeks	↑ hard callus elastic modulus
Topal et al. (2020)	rat	fracture healing	BioMedsa SDÜ Teknokent, Turkey	square	*B* = 0.8 mT, *f* = 7.3 Hz, *DC* = 50%	1 h/day, 4 weeks	↑ bone formation ↓ CTx in serum
van der Jagt et al. (2012)	rat	osteoporosis	- custom-made - Orthopulse (IMD, Netherlands)	square	*- B* = 0.1 mT; *f* = 7.5 Hz; *t* = 0.3 ms *- B* = 0.1 mT, *f* = 15 Hz, 5 ms bursts, *t* = 5 µs	2 h/day, 5 days/week, 6 weeks	-
(Zhou et al., 2013, p. 201)	rat	osteoporosis	XT-2000B (Tianjin xtmed Co., China)	triangular	*B* = 3.8 mT, *f* = 8 Hz, *t* = 0.2 ms	40 min/day, 5 days/week, 12 weeks	↑ serum 17b-estradiol ↓ serum tartrate-resistant acid phosphatase ↑ bone mineral density ↓ deterioration
Wang et al. (2022)	mouse	osteoporosis	custom-made	square	*B* = 2.4–2.6 mT, *f* = 15 Hz, *DC* = 50%	1 h/day, 8 weeks	↑trabecular bone ↑ Osterix, PDGFB and Col-1a1 expression
Li et al. (2017)	mouse	osteoporosis	custom-made	square	*B* = 2 mT, *f* = 15 Hz, 5 ms bursts, *t* = 0.2 ms	2 h/day, 12 weeks	↑ bone formation ↑ serum OCN ↑Wnt/β-catenin signaling
Lei et al. (2017)	mouse	osteoporosis	custom-made	filtered uniform white noise	- LP: low-pass filtered white noise (*f* = 1–100 Hz), *B*_*rms*_ = 0.6 mT - BP: band-pass filtered white noise (*f* = 100–3,000 Hz), *B*_*rms*_ = 1.5 mT - HP: high-pass filtered white noise (*f* = 3,000–50000 Hz) *B*_*rms*_ = 2.5 mT - AP: unfiltered (all-pass) white noise (*f* = 1–50000 Hz) *B*_*rms*_ = 4.5 mT	3 h/day, 8 weeks	LP and BP: ↑ serum bone formation markers ↑ osteogenesis-related gene expressions ↑ bone resorption ↑ RANKL/OPG ratio
Lei et al. (2018)	mouse	osteoporosis	custom-made	square	*B*_*rms*_ = 1.6 mT, *f* = 15 Hz, 5 ms bursts, *t* = 0.2 ms	8 h/day, 8 weeks	↓ deterioration ↑ mechanical properties ↑ Wnt/β-catenin signaling, RANKL, OPG
Androjna et al. (2021)	rat	osteoporosis	custom-made	sinusoidal	*B* = 0.41, 1.2, 4.1, 12.4 mT, *f* = 15 Hz, 5 ms bursts, *t* = 0.26 ms	3 h/day, 6 weeks	1.2 mT: ↓ trabecular bone loss
Wang et al. (2021)	mouse	osteoporosis	custom-made	square	*B* = 1.6 mT, *f* = 8, 50, 75 Hz, *DC* = 50%	1 h/day, 4 weeks	50, 75 Hz: ↓ deterioration, osteoclast numbers ↑ ALP, OCN, Runx2 expression ↓ CTSK, NFATc1, TRAP, CTX-I, IL-1β expression
Jing et al. (2014)	rat	disuse osteopenia	custom-made	square	*B* = 2.4 mT, *f* = 15 Hz, 5 ms bursts, *t* = 0.2 ms	2 h/day, 4 weeks	↓ deterioration ↑ mineral apposition rate, bone formation rate, osteoblast numbers ↑ Wnt1, b-catenin and OCN expressions
Li et al. (2018)	rat	disuse osteopenia	custom-made	square	*B* = 0.6 mT, *f* = 50 Hz, *DC* = 50%	1.5 h/day, 4 weeks	↑ bone mineral density, bone thickness ↑ sAC/cAMP/PKA/CREB signaling
Lin et al. (2020)	mouse	osteopenia	custom-made	square	*B* = 1.8 mT, *f* = 15 Hz, 5.46 ms bursts, *t* = 0.2 ms	8 h/day, 12 weeks	↓ bone loss ↑ bone volume
Sung et al. (2021)	rat	bone growth	custom-made	square	*B* = 2 mT, *f* = 28 Hz, *DC* = 50%	10 h/day, 10 days	↑ growth plate length ↑ circulating IGF-1

As regards the treatment of bone fractures, in 1974 Bassett and Pawluk conducted the first pioneering study, in which pulsing electromagnetic fields have been inductively coupled to dog bone, resulting in improved organization and increased strength of the repaired tissue after fracture (Andrew et al., 1974). Subsequent experiments further validated these findings (Bassett et al., 1977; 1978), ultimately leading in 1979 to the first pre-market approval by the FDA for a PEMF stimulator designed for fracture healing (Orthopaedic and Rehabilitation Devices Panel, 2020). This milestone spurred extensive development of PEMF devices and broadened the clinical application of PEMF stimulation for treating fracture healing. More recently, Bao et al. explored the so-called combined magnetic field (CMF) stimulation, combining static magnetic field and PEMF stimulations. In detail, they implanted intramedullary magnets in rabbit femurs to treat osteotomies and then exposed the animals to PEMF stimulation (*B* = 8–10 mT, *f* = 20 Hz, for 1 h/day) for 5 weeks. The CMF group exhibited increased bone volume and improved trabecular structure, at faster rates, in comparison to both the control and the PEMF-only treated groups (Bao et al., 2019). In 2021, Liu et al. investigated the effect of different intensities of PEMF stimulation (*B* = 1, 5, 10 mT), on rats with osteotomized femurs, showing that higher magnetic field intensity values led to better regeneration outcomes. Namely, rats that received 5 and 10 mT pulses presented higher release of Ca and ALP in the serum, as well as increased bone density, maximum load, fracture load, elastic load, and bending energy of the callus compared to rats exposed to 1 mT (Liu et al., 2021).

Recently, PEMF stimulation has also been explored as a potential adjuvant therapy to enhance implant osseointegration. In case of rabbit bone defects treated with titanium implants, it was reported that PEMF stimulation (*B* = 2 mT, *f* = 15 Hz, square waveform, for 2 h/day for either 6 or 12 weeks) resulted in enhanced bone formation around the implants and upregulated gene expressions in the femoral region, including RUNX2, BMP2, OCN, and Wnt/β-catenin signaling (Jing et al., 2016). In a subsequent study, the same PEMF protocol was delivered for 8 weeks to rabbits with type 1 diabetes and porous titanium implants. The PEMF stimulation mitigated bone deterioration and promoted both osseointegration and bone ingrowth into implant pores, reducing bone loss by activating Wnt/β-catenin signaling (Cai et al., 2018). Concerning the stability of the implant, Nunes et al. tested on rats with titanium implants the effects of two different PEMF exposure regimens (1 and 3 h/day, *B* = ± 1 mT, *f* = 15 Hz, triangular waveform). A peculiar dependence on the exposure duration was observed, as 1 h/day resulted in better outcomes in removal torque tests, bone volume and mineral density, cell viability, total protein content, and mineralization nodules in comparison to the 3 h/day regimen, while this latter protocol yielded higher trabecular bone thickness and cell proliferation (Nunes et al., 2021). Adopting a different approach, Barak and co-workers developed a miniaturized PEMF stimulator embedded in a commercially available dental implant, which was implanted in rabbit tibiae. Already after 2 weeks of continuous exposure (*B* = 0.2–0.4 mT, *f* = 10 Hz), test implants showed a significantly higher trabecular bone fraction, enhanced connectivity, and higher bone-to-implant contact as compared to the control group (Barak et al., 2016).

Over the past decade, researchers have also focused on exploring the potential of PEMF stimulation as a treatment for osteoporosis, for which mice or rats have been used as animal models suitable for delivering whole-body treatments. Regarding osteoporotic fractures, in 2014, Androjna and co-workers performed fibular osteotomies on female rats with osteopenia and subjected them to PEMF treatment (*B* = 0.5 mT, *f* = 15 Hz, triangular waveform, for 3 h/day over 6 weeks), observing increased elastic modulus of the hard callus across fibula fractures (Androjna et al., 2014). Moreover, PEMF treatment (*B* = 0.8 mT, *f* = 7.3 Hz, square waveform, for 1 h/day for 28 days) increased new bone formation and reduced bone degradation markers in rats with heparin-induced osteoporosis (Topal et al., 2020). Concerning the direct treatment of osteoporosis, Van der Jagt et al. did not observe significant differences in female rats with ovariectomy-induced osteoporosis when exposing them to different PEMF stimulations (*B* = 0.1 mT, *f* = 15 or 7.5 Hz, *t* = 0.3 or 5 ms burst, square waveform, for 2 h/day for 5 days/week over 6 weeks) in comparison to unstimulated controls (van der Jagt et al., 2012). However, adopting the same animal model but using different PEMF parameters with higher B values (i.e., *B* = 3.8 mT (Zhou et al., 2013); *B* = 2.4–2.6 mT (Wang et al., 2022)), significant improvements were observed in bone mineral density, trabecular bone amount, serum 17β-estradiol levels, and inhibition of bone microarchitecture deterioration. Recently, the application of PEMF (*B* = 2 mT, *f* = 15 Hz, square waveform, for 3 h/day) on diabetic osteoporotic mice was demonstrated to enhance bone formation through upregulation of osteoblastogenesis-related genes via the Wnt/β-catenin pathway, while no significant effects on osteoclastogenesis-related genes were observed (Li et al., 2017). Similarly, Lei and colleagues demonstrated that exposing ovariectomized mice to PEMF stimulation (*B* = 0.6 and 1.5 mT, *f* = 1–3,000 Hz, for 3 h/day over 8 weeks) significantly increased bone formation markers and osteogenic gene expression, while higher B values (*B* = 2.5 and 4.5 mT, *f* = 3,000–50,000 Hz) reduced these effects. Additional findings showed improved trabecular architecture and mechanical properties, with upregulation of Wnt/β-catenin, RANKL, and OPG, though the RANKL/OPG mRNA ratio remained unchanged (Lei et al., 2017; 2018). Further studies focused on the impact of different magnetic field intensity and frequency values. Namely, in 2021, Androjna and co-workers compared four different PEMF protocols (*B* = 0.41, 1.2, 4.1, and 12.4 mT, *f* = 15 Hz, sinusoidal waveform, for 3 h/day over 6 weeks) with the efficacy of the alendronate drug, used to prevent or treat osteoporosis. The authors found that PEMF stimulation with *B* = 1.2 mT was nearly as effective as alendronate in reducing trabecular bone loss. Moreover, PEMF with *B* = 1.2 mT and 4.1 mT altered lacuno-canalicular features, suggesting osteocyte sensitivity to PEMF (Androjna et al., 2021). In the same year, Wang et al. applied to ovariectomized mice PEMF stimulation (*B* = 1.6 mT, square waveform, for 1 h/day over 4 weeks) at different frequencies (*f* = 8, 50, 75 Hz). At high frequencies (i.e., 50 and 75 Hz), they observed improved bone microarchitecture, decreased osteoclast numbers, promoted osteoblast-related gene expression (ALP, OCN, RUNX2), and inhibited osteoclast-related genes (CTSK, NFATc1, TRAP) and bone resorption markers like CTX-I and IL-1β (Wang et al., 2021).

Since osteopenia and osteoporosis can also result from disuse conditions, such as prolonged bed rest, immobilization after injury, or exposure to altered gravity in space travel, the potential of PEMF stimulation as a treatment for bone loss was investigated. In 2014, Jing et al. revealed that applying PEMF stimulation (*B* = 2.4 mT, *f* = 15 Hz, square waveform, for 2 h/day over 4 weeks) to hindlimb-unloaded rats prevented the deterioration of bone microarchitecture and promoted bone formation, with increased expression of Wnt1, β-catenin, and osteocalcin genes (Jing et al., 2014). Similarly, it was reported that PEMF treatment (*B* = 0.6 mT, *f* = 50 Hz, square waveform, for 1.5 h/day for 4 weeks) helped to maintain bone mineral density and cortical bone thickness in suspended rats, likely through activation of the sAC/cAMP/PKA/CREB pathway (Li et al., 2018). In 2020, Lin et al. induced disuse osteopenia in mice and then applied either PEMF stimulation (*B* = 1.8 mT, *f* = 15 Hz, square waveform, for 8 h/day over 12 weeks) or single-pulsed electromagnetic field (SPEMF) stimulation (*B* = 1 T, *f* = 0.2 Hz, asymmetrical half-sine waveform, for 3 min/day over 12 weeks). SPEMF significantly reversed bone loss as early as 6 weeks post-treatment, while PEMF reversed bone loss after 8 weeks, with a significant increase in bone volume for both groups in comparison to unstimulated mice (Lin et al., 2020). Finally, the effect of PEMF on bone growth without the presence of any altered condition was investigated by Sung et al., in 2021, exposing rats to PEMF stimulation (*B* = 2 mT, *f* = 28 Hz, square waveform, for 10 h/day along 10 days) and then analyzing the animals’ tibiae. In treated rats, the length of the growth plate resulted significantly higher, as well as the levels of circulating IGF-1 (Sung et al., 2021).

Overall, these studies suggest that PEMF can promote bone health by modulating osteogenesis and bone resorption pathways across various pathological conditions, including implant osseointegration, osteoporosis, and inactivity-related bone loss. However, the PEMF efficacy depends on specific parameters, emphasizing the need for further research to optimize therapeutic protocols.

### 6.2 Quantitative comparison among *in vivo* studies on PEMF stimulation for bone tissue

**FIGURE 5:**
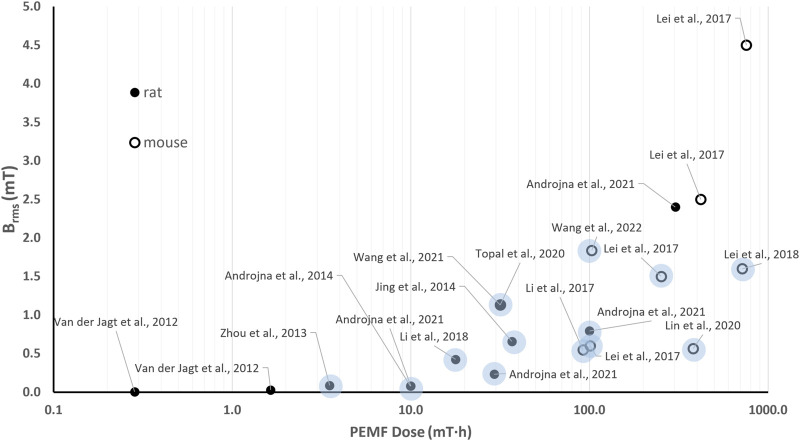
Distribution of the root mean square value of the magnetic field intensity (*B*_*rms*_) and the PEMF dose (*D*) value for the *in vivo* studies performed imposing PEMF stimulation on bone tissue. The light dots refer to studies reporting positive effects of PEMF stimulation on pathological conditions related to osteoporosis. The different symbols refer to different animal models.

The *in vivo* investigations of the effects of PEMF stimulation on bone tissue report a wide range of PEMF stimulation parameters (*B* = 0.1–12.4 mT, *f* = 1–100 Hz), with studies lasting from 10 days to 12 weeks and exposure durations ranging from 40 min/day to 10 h/day. Therefore, this results in *B*_*rms*_ values ranging from 0.005 to 4.5 mT and *D* values ranging from 2∙10^–2^–10^3^ mT·h. Figure 5 shows the distribution of the *B*_*rms*_ and *D* values for the *in vivo* experiments focusing on the use of PEMF for the treatment of pathological conditions related to osteoporosis, such as osteoporotic fractures, disuse osteopenia, and osteoporosis.

Interestingly, regarding the use of PEMF for the treatment of osteoporosis-related pathologies, all the reported studies delivered whole-body PEMF stimulation to either rats or mice, enhancing the consistency of the proposed comparison. In particular, no significant PEMF effects were observed on rat osteoporotic bone when low-intensity PEMF (*B* = 0.1 mT) was imposed, corresponding to *B*_*rms*_ values equal to 0.005 mT (*f* = 7.5 Hz, *t* = 0.3 ms) and 0.027 mT (*f* = 15 Hz, burst duration = 0.5 ms) for the two tested groups (van der Jagt et al., 2012). The received PEMF doses (*D* = 0.29 mT·h and *D* = 1.65 mT·h, respectively) proved insufficient to trigger a response. Adopting the same animal model, Zhou and co-workers applied a higher peak intensity (*B* = 3.8 mT, *f* = 8 Hz) and found positive effects of PEMF stimulation in terms of inhibited deterioration of bone microarchitecture (Zhou et al., 2013). Indeed, although the applied stimulation resulted in a low *B*_*rms*_ value (0.088 mT), this was for a longer exposure duration, leading to a higher total PEMF dose equal to 3.51 mT·h. Two comparative studies, conducted independently in 2017 (Lei et al., 2017) and in 2021 (Androjna et al., 2021), offer further insights on the correlation between the applied *B*_*rms*_ and *D* and the biological effects. In detail, Lei et al. subjected ovariectomized osteoporotic mice to 4 different PEMF stimulation protocols, based on uniform white noise filtered at different frequencies, for 3 h/day over 8 weeks. Interestingly, only two stimulation protocols, resulting in *B*_*rms*_ values of 0.6 mT and 1.5 mT and PEMF dose values of 100.8 and 252 mT·h, respectively, significantly increased serum bone formation markers and osteogenesis-related gene expressions in comparison to controls. The other tested protocols, characterized by higher *B*_*rms*_ and *D* values, did not prove as effective (Lei et al., 2017). In parallel, Androjna and co-workers compared four different PEMF protocols (*B* = 0.41, 1.2, 4.1, and 12.4 mT, *f* = 15 Hz, sinusoidal waveform, for 3 h/day over 6 weeks) with the efficacy of the alendronate drug (Androjna et al., 2021). The most effective protocols were characterized by *B*_*rms*_ values equal to 0.23 and 0.79 mT and *D* values equal to 29.3 mT·h and 100 mT·h, respectively. The other protocols, characterized by the lowest *B*_*rms*_ value (0.08 mT) and *D* equal to 10 mT·h or by the highest *B*_*rms*_ value (2.4 mT) with *D* = 302.6 mT·h, were not effective.

Notably, most of the studies that reported positive effects of *in vivo* PEMF stimulation on pathological conditions related to osteoporosis (Jing et al., 2014; Li et al., 2017; Lei et al., 2018; Lin et al., 2020; Topal et al., 2020; Wang et al., 2021) are characterized by *B*_*rms*_ values lower than 1.84 mT and *D* values higher than 3.51 mT·h (light blue dots in Figure 5). When higher *B*_*rms*_ values were provided, the corresponding protocols did not prove effective, regardless of the high PEMF dose associated (Lei et al., 2017; Androjna et al., 2021).

### 6.3 Cartilage and osteochondral tissue

The promising results of *in vitro* application of PEMF on cartilage regeneration have prompted investigations into its *in vivo* effects, with a particular focus on cartilage degeneration and OA in different articular districts (see Table 7). As regards the intervertebral disc, in 2014, rats with surgically induced degeneration were treated with PEMF imposing the same magnetic field intensity (*B* = 5 mT, square waveform) and two different frequencies (*f* = 10, 100 Hz) for 2 h/day over 12 weeks. Both PEMF protocols attenuated intervertebral degenerative processes, with a significant decrease of Cleaved caspase-3 and Bax/Bcl-2 ratio compared to the untreated group (Reihani Kermani et al., 2014). Many more studies investigated PEMF impact on knee articular defects. In 2011, Li and co-workers exposed rats with ovariectomy-induced knee cartilage apoptosis to PEMF stimulation (*B* = 3.8 mT, *f* = 8 Hz, for 40 min/day along 30 days), observing significantly upregulated X-linked inhibitor of apoptosis (XIAP) mRNA and estrogen and downregulated Bax in knee joint cartilage (Li et al., 2011). In 2015, Veronesi et al. created surgical lesions in both knees of 10 rabbits and implanted a collagen scaffold in one lesion and a collagen scaffold seeded with bone marrow cells in the other. Afterwards, they provided PEMF stimulation (*B* = 1.5 mT, *f* = 75 Hz, quasi-triangular waveform, for 4 h/day over 40 days) on half of the animals. PEMF alone improved both cartilage cell and matrix parameters compared with scaffold alone. Additionally, the combination of cell-seeded scaffold and PEMF further improved osteochondral regeneration in terms of cartilage cellularity, matrix parameters, and reduced percentage of cartilage under the tidemark (Veronesi et al., 2015). A similar approach was adopted in 2020, when engineered osteochondral constructs were implanted in focal cartilage defects created in the stifle joints of eight dogs. After 3 months of PEMF stimulation (*B* = 1.5 mT, *f* = 75 Hz, quasi-triangular waveform, for 6 h/day), treated animals were less likely to have proteoglycan- and chondrocyte-related pathology than control, with tissue-engineered repair integration improved by PEMF, although not significantly (Stefani et al., 2020). Recent studies confirmed that PEMF can inhibit cartilage degradation, reducing the upregulation of pro-inflammatory and degradative factors in synovium (Liu et al., 2022; Ma et al., 2024).

**TABLE 7:** *In vivo* studies on the effects of PEMF stimulation on cartilage and osteochondral tissue.

Reference	Species	Investigated condition	PEMF stimulator	PEMF waveform	PEMF parameters	PEMF exposure	Main biological outcomes/Signaling pathways involved
Reihani Kermani et al. (2014)	rat	intervertebral discs degeneration	Fisiofield Mini (Fisioline Co., Italy)	square	*B* = 5 mT, *f* = 10–100 Hz, *t* = 5–50 ms	2 h/day, 12 weeks	↓ degeneration ↓ Cleaved caspase-3, Bax/Bcl-2 ratio
Li et al. (2011)	rat	apoptosis modulation	Union-2000 (Chinese Academy of Medical Sciences, China)	-	*B* = 3.8 mT, *f* = 8 Hz	40 min/day, 30 days	↑ XIAP mRNA, estrogen ↓ Bax
Veronesi et al. (2015)	rabbit	osteochondral regeneration	I-ONE (IGEA, Italy)	quasi-triangular	*B* = 1.5 mT, *f* = 75 Hz, *t* = 1.3 ms	4 h/day, 40 days	↑ cartilage cellularity, matrix parameters ↓ percentage of cartilage under the tidemark
Stefani et al. (2020)	dog	implant integration	IGEA, Italy	quasi-triangular	*B* = 1.5 mT, *f* = 75 Hz, *t* = 1.3 ms	6 h/day, 3 months	↑ likelihood of normal chondrocyte ↑ proteoglycan histological scores
Liu et al. (2022)	rat	osteoarthritis	custom-made	-	*B* = 3.82 mT, *f* = 8 Hz	40 min/day, 5 days/week, 12 weeks	↓ inflammation, degeneration ↓ NLRP3, Caspase-1, GSDMD expression
Ma et al. (2024)	rat	osteoarthritis	GHY-III, FMMU, Xi’an, China	triangular	*B* = 2 mT, *f* = 15 Hz, 5 ms bursts, *t* = 0.2 ms	2 h/day, 6 weeks	↓ inflammation, degeneration
Veronesi et al. (2014)	guinea pig	osteoarthritis	IGEA, Italy	quasi-triangular	*B* = 1.5 mT, *f* = 37–75 Hz, *t* = 1.3 ms	6 h/day, 3 months	↓ histological cartilage score, trabecular number ↑ trabecular thickness
Zhou et al. (2017)	rat	osteoarthritis	Hunan Forever Elegance Technology Co., Ltd., China	-	*B* = 8 mT, *f* = 20 Hz	40 min/day, 5 days/week, 12 weeks	↓ MAPKs signaling
Yang et al. (2017)	rat	osteoarthritis	custom-made	sinusoidal	*B* = 1.6 mT, *f* = 75 Hz, *t* = 1.3 ms	2 h/day, 4 weeks	↓ (moderate) cartilage degradation
Ye et al. (2020)	mouse	osteoarthritis	custom-made	square	*B* = 1.6 mT, *f* = 75 Hz, *DC* = 50%	1 h/day, 4 weeks	↑ bone volume fraction, trabecular thickness, trabecular number ↑ ACAN expression ↓ IL-1β, ADAMTS4, MMP13 expressions
Yang et al. (2021)	mouse	osteoarthritis	custom	square	*B* = 3.8 mT, *f* = 75 Hz, *DC* = 50%	1 h/day, 4 weeks	↓ pain, degeneration, synovitis ↓ IL-6, TNF-α expression

Regarding the direct treatment of OA, Veronesi and colleagues exposed guinea pigs with OA to PEMF stimulation (*B* = 1.3 mT for 6 h/day over 3 months) imposing two different frequencies (*f* = 37 or 75 Hz). PEMF significantly reduced histological cartilage score, fibrillation index, subchondral bone thickness, and trabecular number while increased trabecular thickness and separation in comparison to the untreated group. Moreover, stimulation at 75 Hz significantly improved the histological score (Veronesi et al., 2014). In 2017, Zhou et al. treated an experimental rat model of OA with PEMF stimulation (*B* = 8 mT, *f* = 20 Hz, for 40 min/day for 5 days/week over 12 weeks) and observed partially prevented cartilage destruction, with inhibition of MAPKs signaling pathway (Zhou et al., 2017). In parallel, Yang and coworkers investigated the effect of the time of delivery of PEMF stimulation (*B* = 1.6 mT, *f* = 75 Hz, for 2 h/day over 4 weeks) on rats with induced knee OA, finding that timing is crucial. Pre-emptive PEMF maintained the microarchitecture of subchondral trabecular bone and partial chondroprotective properties were observed in pre-emptive and early PEMF treatment groups (Yang et al., 2017). Recently, the efficacy of PEMF for treating OA was also compared to the whole-body vibration treatment in mice with knee OA. Both PEMF stimulation (*B* = 1.6 mT, *f* = 75 Hz, square waveform, for 1 h/day) and vibration treatment (frequency = 5 Hz, amplitude = 4 mm, gravitational acceleration = 0.3 g, for 20 min/day) were applied over 4 weeks, leading to increased bone volume fraction, trabecular thickness, and trabecular number. In addition, the expression of ACAN was promoted, while the surface to volume ratio of bone was reduced, complemented by inhibited expressions of inflammatory cytokines interleukin-1 (IL-1β), and downregulated expression of the catabolic factor MMP13, with better results observed in PEMF-treated mice (Ye et al., 2020). Based on these results, in 2021, the abovementioned animal models were treated with PEMF with increased magnetic field intensity (*B* = 3.8 mT), resulting in a beneficial effect on pain, cartilage degeneration, synovitis, and trabecular bone microarchitecture. Moreover, PEMF slowed the structural and functional progression of OA by inhibiting TNF-α and IL-6 signaling and ameliorated cartilage matrix, reducing chondrocyte apoptosis and autophagy (Yang et al., 2021).

In summary, it has been demonstrated that PEMF treatment can improve cartilage health, enhance matrix parameters, and inhibit degenerative processes in osteochondral tissue. In addition, PEMF can modulate inflammatory pathways (Supplementary Table S1), reduce cartilage destruction, and improve bone and joint health in OA models. These findings suggest that PEMF may be an effective non-invasive treatment for OA, particularly when applied at specific intensities and frequencies.

### 6.4 Quantitative comparison among *in vivo* studies on PEMF stimulation for cartilage and osteochondral tissue

**FIGURE 6:**
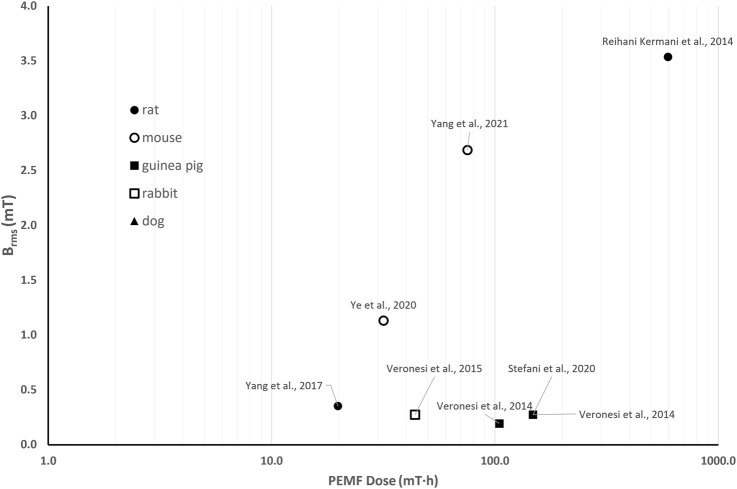
Distribution of the root mean square value of the magnetic field intensity (*B*_*rms*_) and the PEMF dose (*D*) value for the *in vivo* studies performed imposing PEMF stimulation on cartilage and osteochondral tissues. The different symbols refer to different animal models.

Besides variations in the intensity of the imposed magnetic field (resulting in *B*_*rms*_ values ranging from 0.19 to 5.66 mT, Figure 6), *in vivo* studies on PEMF stimulation investigating its effects on cartilage and osteochondral repair are distinguished by PEMF dose values ranging from 19.8 to 594 mT·h. All studies reported the attenuation of degenerative processes in cartilage via the inhibition of pathways related to inflammatory response and apoptosis. Interestingly, the study on guinea pigs with OA exposed to PEMF stimulation adopting two different frequency values (*B* = 1.3 mT, *f* = 37 or 75 Hz) found positive effects with both protocols, but better histological score was obtained when the highest frequency was used, which also corresponds to the highest PEMF dose (*D* = 104.6 mT·h for *f* = 37 Hz and *D* = 147.9 mT·h for *f* = 75 Hz) (Veronesi et al., 2014). However, given the limited number of available studies, no clear correlations between PEMF parameters and biological responses can be established from this analysis.

## 7 Concluding remarks

Over the past three decades, due to its non-invasiveness and promising outcomes, PEMF stimulation has gained widespread adoption as a clinical intervention for enhancing the treatment of various bone and cartilage disorders, including non-union bone fractures, osteoporosis, and OA (Haddad et al., 2007; Court-Brown et al., 2017). Additionally, PEMF has garnered attention as a potential countermeasure for mitigating the adverse effects of disuse conditions, including those caused by altered gravity during space travel, as evidenced by two NASA patents (Goodwin and Parker, 2007; Goodwin and Shackelford, 2014). However, despite the increasing availability of PEMF devices on the market and their growing use in clinical practice, the PEMF stimulation parameters are largely applied empirically with a persistent lack of standardization (Yuan et al., 2018). As illustrated in Table 2, there is substantial heterogeneity among commercially available PEMF stimulators, with significant variation in key parameters. Moreover, the absence of standardization has led to a multitude of studies, both *in vitro* (Tables 4, 5) and *in vivo* (Tables 6, 7), with the aim of elucidating the biological phenomena induced by PEMF at cellular and tissue levels.

However, this variability in experimental set-ups and imposed PEMF parameters complicates the direct comparison of outcomes across studies. This challenge is further compounded by inconsistencies in device specifications and experimental protocols, which introduce confounding factors that obscure the interpretation of results. Moreover, the lack of tunability in the parameters of most commercial devices imposes limitations on the capacity of research groups to conduct comparative studies, to vary the stimulation conditions, and to replicate the set-ups adopted by other researchers. Consequently, for the advancement of PEMF research, it would be highly desirable to adopt, at least in the early stages, standardized stimulation parameters to facilitate comparability across studies. Although standardization remains a significant challenge in the field, it is noteworthy that a limited number of research efforts have followed a rigorous translational approach, consistently applying identical PEMF parameters across *in vitro* (Saino et al., 2011; Ongaro et al., 2012), *in vivo* (Veronesi et al., 2015; Stefani et al., 2020), and clinical investigations (Massari et al., 2006). This methodological coherence not only strengthens the reliability of the findings but also supports the generation of clinical evidence with greater translational relevance. These examples demonstrate that it is indeed feasible to design and implement protocols that maintain parameter fidelity throughout the translational pipeline, ultimately contributing to the development of clinically validated therapeutic applications.

To facilitate the comparability of past and future research, here we proposed a quantitative approach based on the calculation of the PEMF dose—a comprehensive metric that integrates magnetic field intensity, stimulation waveform and exposure duration. Thanks to this method, we were able to highlight some common outcomes among different studies. Concerning bone tissue, from the comparison of the *in vitro* studies (Figure 3) we concluded that, for low *B*_*rms*_ values (<0.3 mT), the exposure duration plays a crucial role and a minimum PEMF dose of 10 mT∙h should be guaranteed for promoting osteogenic differentiation. While, when higher magnetic field intensities (*B*_*rms*_ ≥ 0.3 mT) are provided, a lower minimum PEMF dose threshold (*D* ≥ 0.21 mT∙h) is required to induce osteogenic effects. Comparing the *in vivo* animal studies on osteoporosis (Figure 5), the best results were obtained exploiting *B*_*rms*_ values below 1.84 mT and *D* values above 3.51 mT·h (light blue dots in Figure 5). Regarding cartilage tissue, *in vitro* investigations suggest that the PEMF dose is a pivotal factor in determining whether the PEMF stimulation favors cell proliferation or differentiation (Figure 4). Specifically, it was observed that with *D* ranging from 3.5 to 26 mT·h, cell proliferation was induced (yellow dots in Figure 4), while at PEMF doses exceeding 26.02 mT·h, a shift towards chondrogenic differentiation was promoted (light blue dots in Figure 4). Concerning *in vivo* studies, given the low number of studies available in literature, the proposed quantitative approach did not lead to the extraction of significant evidence (Figure 6).

The method proposed for quantitative comparison is affected by some limitations. In the PEMF dose calculation, the frequency and pulse duration parameters are combined in the duty cycle and do not account separately, although the adopted frequency could be an important factor to consider for osteogenic differentiation (Luo et al., 2012; Poh et al., 2018). Additionally, only studies that explicitly reported all relevant PEMF parameters were included in the PEMF dose calculation for the quantitative comparison, as the absence of such data precludes PEMF dose estimation. Furthermore, the proposed approach does not account for cell type or animal models, factors that could influence the observed effects of PEMF stimulation. Despite these limitations, the synthesized findings provide valuable insights that can guide the refinement of future research on the biological effects of PEMF stimulation.

For the future of PEMF research, it would be desirable to use, at least initially, the same parameters to ensure better comparability. Subsequently, it would make sense to systematically vary the various parameters individually until the best possible combination is achieved (Flatscher et al., 2023). To support parameter selection for comparative studies and assist in the design of new experiments, the proposed log-log graph of *B*_*rms*_ versus *t*_*exp*_ (Figure 2) represents a valuable tool. Indeed, by displaying PEMF dose values as straight isolines and incorporating the *B*_*rms*_ value of a given PEMF stimulator, this graphical approach enables researchers to determine the corresponding *t*_*exp*_ values relevant for future investigations. Log-log graphs of *B*_*rms*_ versus *t*_*exp*_ for the studies analyzed in the quantitative sections are provided in the Supplementary Material (Supplementary Figures S1–S4). Moreover, in order to perform more realistic *in vitro* studies, the use of 3D biomimetic tissue models cultured in bioreactors could offer significant potential to refine experiments and overcome the limitations of the conventional static 2D cultures. In the reviewed literature, only three studies have used 3D *in vitro* tissue models cultured in bioreactors (Wang H. et al., 2019; Gabetti et al., 2022; Daou et al., 2024), although the results suggest that bioreactors could represent powerful tools for in depth investigating the cell and tissue behavior under PEMF treatment. Furthermore, adopting more descriptive and reliable models would enable the refinement of the tested conditions and the reduction of the number of animals for the *in vivo* experiments, which often fail to provide directly translatable predictions for human outcomes. This approach also aligns with the 3R principles, promoting more ethical and efficient research methodologies.

In conclusion, this review provides a comprehensive overview of the current state of the art of *in vitro* and *in vivo* experiments investigating the biological effects of PEMF stimulation on bone and cartilage, underscoring the great variability in the models and conditions employed across studies. By introducing a method for quantitatively comparing the reviewed studies, we aim to foster the refinement of future research and support the development of standardized guidelines for PEMF treatment. Future investigations should prioritize the identification of optimal PEMF parameters, including amplitude, frequency, waveform, and exposure duration, through harmonized and reproducible methodologies. Establishing consensus on experimental models, outcome measures, and stimulation protocols is essential to improve comparability across studies and to generate robust, translatable data. These would be crucial for deepening the understanding of cellular responses and for establishing optimal PEMF treatment protocols to promote effective bone and cartilage repair.
