# Supplementary material for: Insights into bone and cartilage responses to pulsed electromagnetic field stimulation: a review with quantitative comparisons
Source: Front Bioeng Biotechnol. 2025 Jul 10;13:1557572. doi: 10.3389/fbioe.2025.1557572 (PMC12287613; doi:10.3389/fbioe.2025.1557572)
Supplement: Supplementary file 1 [file DataSheet1.pdf]

Insights into Bone and Cartilage Responses to Pulsed Electromagnetic Field Stimulation: A Review with Quantitative Comparisons

Beatrice Masante[1,2†], Stefano Gabetti[1,2†], Joao C. Silva[1,2], Giovanni Putame[1,2], Simone Israel [1,2], Cristina Bignardi[1,2], Diana Massai[1,2*]

[1]Department of Mechanical and Aerospace Engineering and Polito^{BIO}Med Lab, Politecnico di Torino, Turin, Italy

[2]Interuniversity Center for the Promotion of the 3Rs Principles in Teaching and Research, Turin, Italy

†These authors contributed equally to this work and share first authorship

***Correspondence:**
Diana Massai
diana.massai@polito.it

Supplementary material

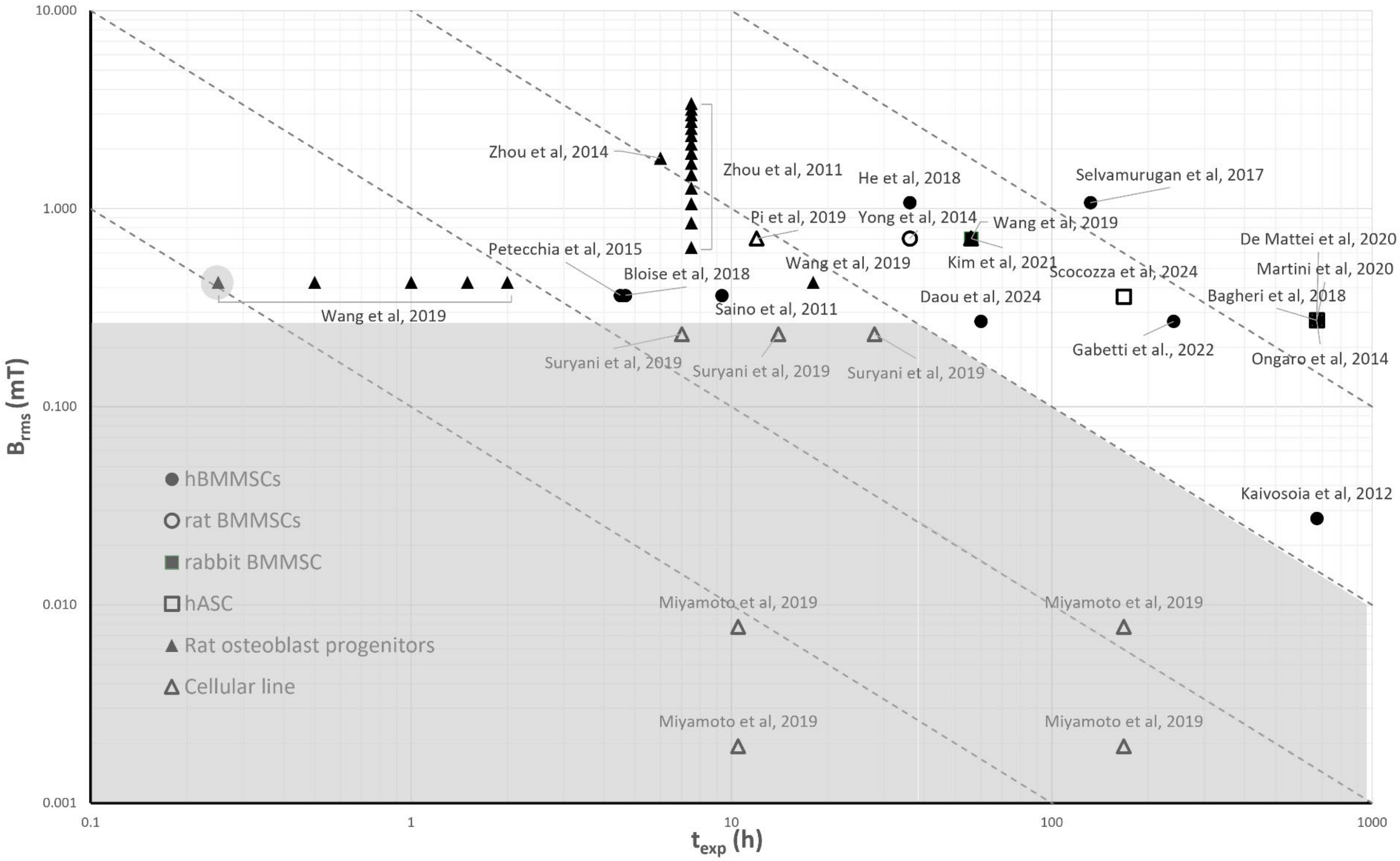

Supplementary Figure 1. Log-log graph of the root mean square value of the magnetic field intensity (B_{rms}) versus the total exposure duration (t_{exp}), for the in vitro studies performed imposing PEMF stimulation on bone cells/tissues. The oblique dashed lines represent the isolines of constant D values. The grey area represents the threshold region for the B_{rms} and D values, within which the likelihood of observing an osteogenic effect induced by PEMF stimulation is low. The different symbols refer to different cell types.

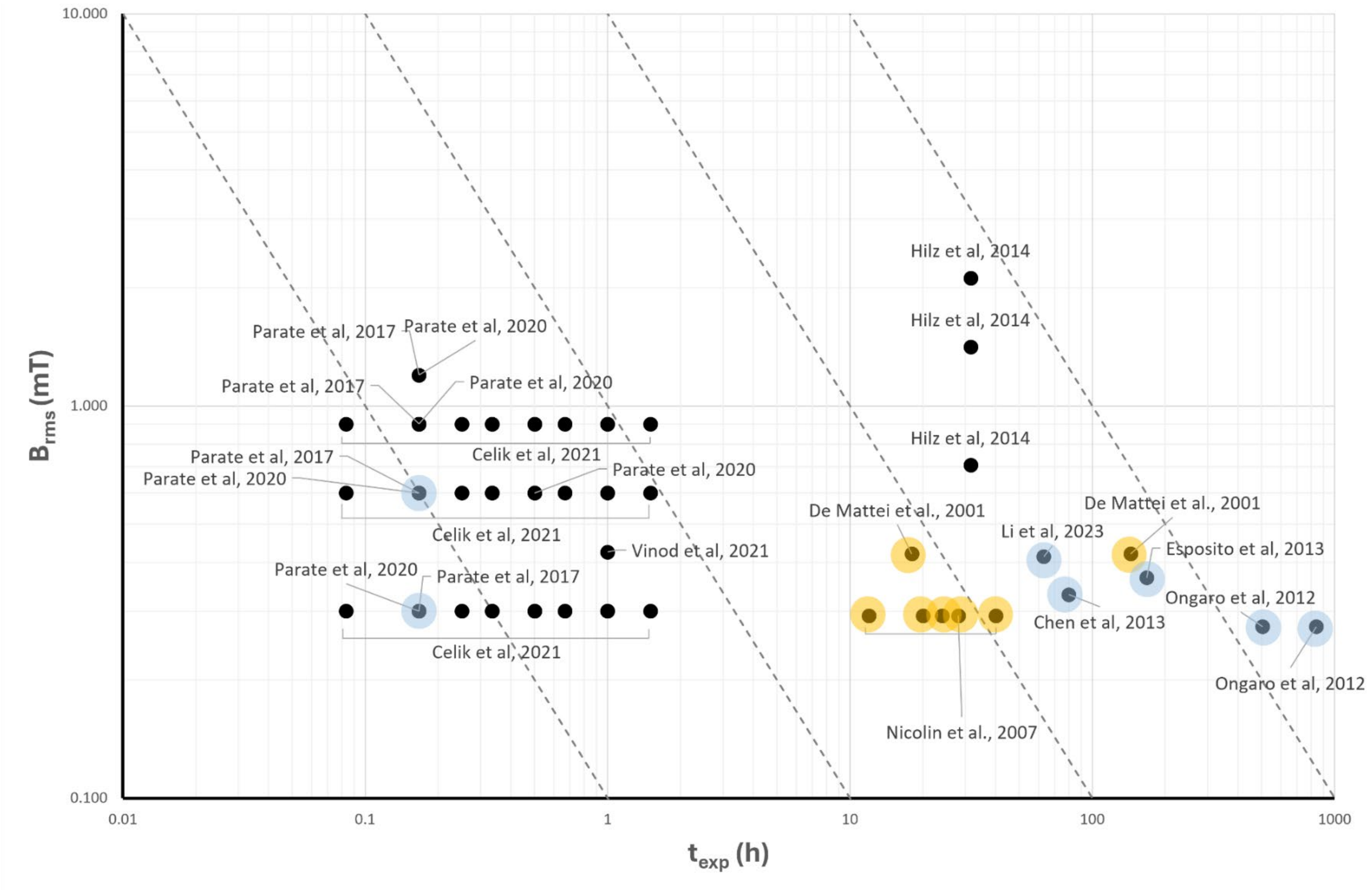

Supplementary Figure 2. Log-log graph of the root mean square value of the magnetic field intensity (B_{rms}) versus the total exposure duration (t_{exp}), for the in vitro studies performed imposing PEMF stimulation on cartilage cells/tissues. The oblique dashed lines represent the isolines of constant D values. The colored dots refer to studies reporting enhanced chondrocyte proliferation (yellow dots) or promoted chondrogenic differentiation (light blue dots).

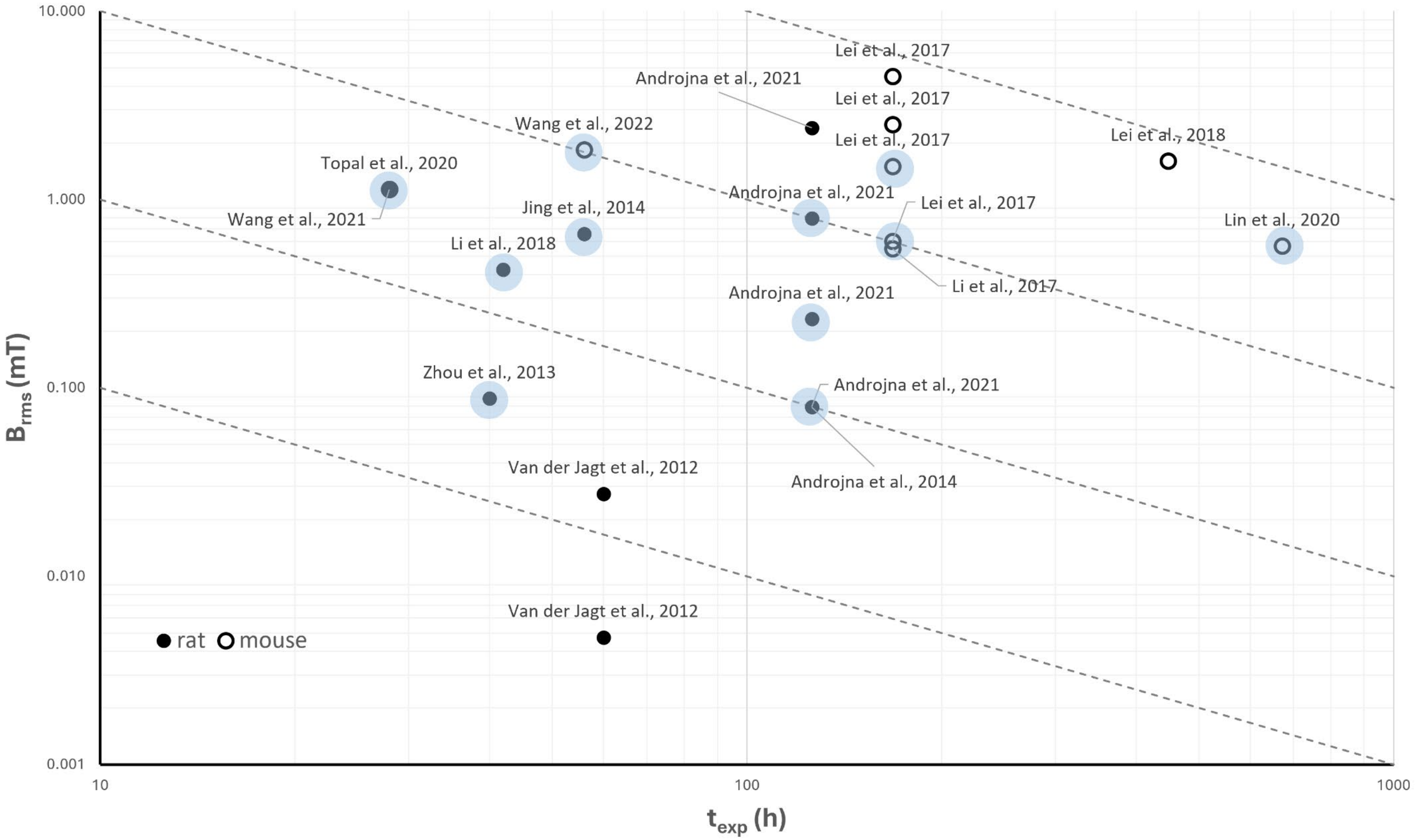

Supplementary Figure 3. Log-log graph of the root mean square value of the magnetic field intensity (B_{rms}) versus the total exposure duration (t_{exp}), for the in vivo studies performed imposing PEMF stimulation on bone tissue. The oblique dashed lines represent the isolines of constant D values. The light dots refer to studies reporting positive effects of PEMF stimulation on pathological conditions related to osteoporosis. The different symbols refer to different animal models.

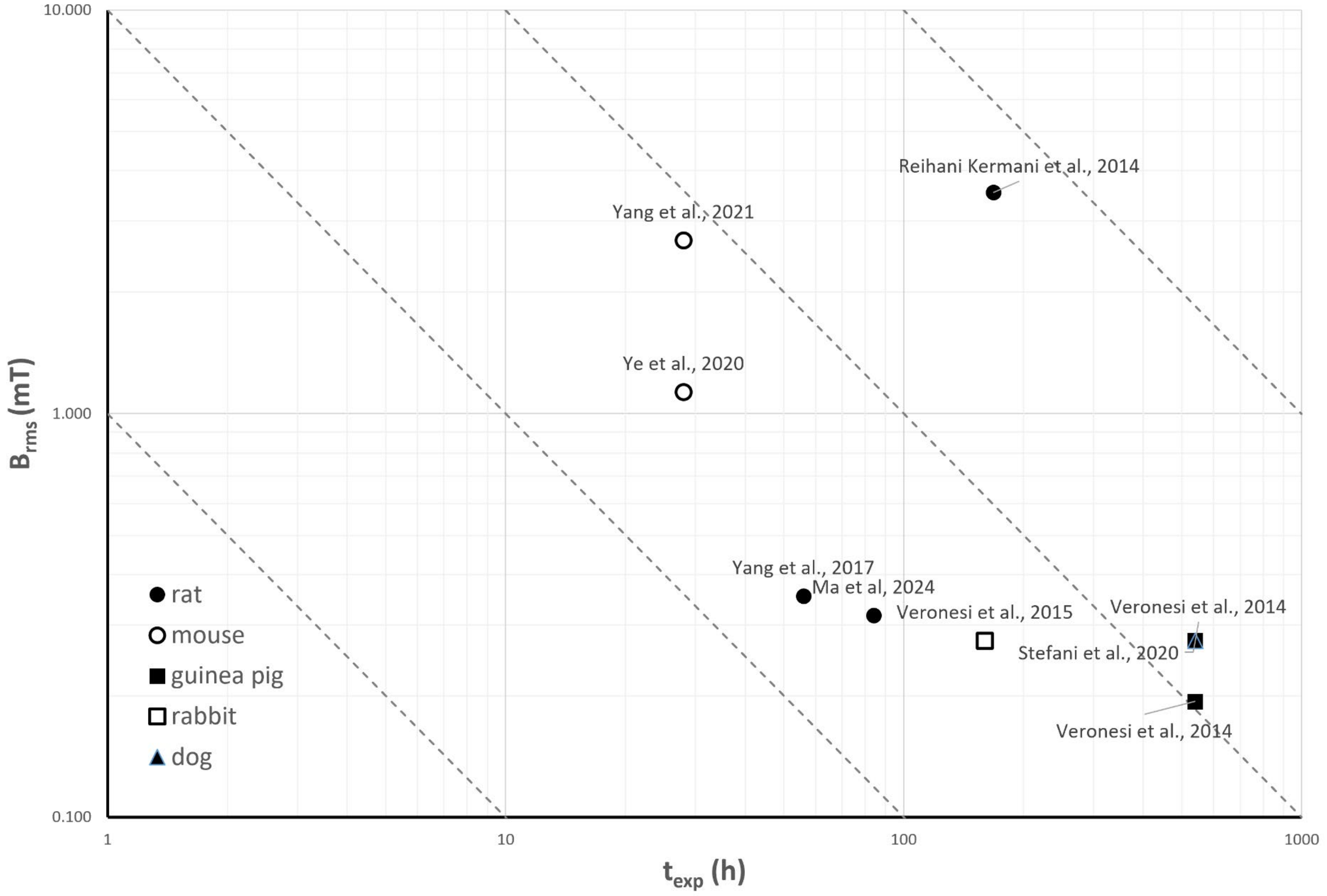

Supplementary Figure 4. Log-log graph of the root mean square value of the magnetic field intensity (B_{rms}) versus the total exposure duration (t_{exp}), for the in vivo studies performed imposing PEMF stimulation on cartilage and osteochondral tissues. The oblique dashed lines represent the isolines of constant D values. The different symbols refer to different animal models.

Supplementary Table 1. Summary of the signaling pathways activated by PEMF stimulation in the context of bone and cartilage regeneration and main related biological responses.

Signaling pathway	PEMF effects	Biological outcome
BMP/Smad	↑ BMP2, ↑ p-Smad1/5/8, ↑ Runx2, ↑ Sox9	Promotes MSC's osteogenic and chondrogenic differentiation
MAPK (ERK, p38)	↑ ERK → proliferation; ↑ p38 → Runx2/Sox9 activation	Supports MSC's viability and differentiation
Wnt/β-Catenin	↑ β-Catenin stability, ↑ osteogenic gene expression	Promotes MSC's osteogenic differentiation
Notch	Modulated (transient activation/inhibition)	Balances MSC's differentiation vs. self-renewal
Calcium/CaM	↑ Intracellular Ca^{2+} → CREB, CaMKII signaling	Enhances mechanotransduction and the expression of osteogenic transcription factors (e.g. Runx2)
TNF-α/IL-6	↓ TNF-α and IL-6	Ameliorate cartilage matrix, reduce chondrocyte apoptosis and autophagy
